# Orally Administered Enoxaparin Ameliorates Acute Colitis by Reducing Macrophage-Associated Inflammatory Responses

**DOI:** 10.1371/journal.pone.0134259

**Published:** 2015-07-28

**Authors:** Qi Ying Lean, Rajaraman D. Eri, Sarron Randall-Demllo, Sukhwinder Singh Sohal, Niall Stewart, Gregory M. Peterson, Nuri Gueven, Rahul P. Patel

**Affiliations:** 1 Pharmacy, School of Medicine, Faculty of Health, University of Tasmania, Hobart, Tasmania, Australia; 2 Faculty of Pharmacy, University of Technology MARA, Puncak Alam, Selangor, Malaysia; 3 School of Health Sciences, Faculty of Health, University of Tasmania, Launceston, Tasmania, Australia; 4 Breathe Well Centre of Research Excellence for Chronic Respiratory Disease and Lung Ageing, School of Medicine, Faculty of Health, University of Tasmania, Hobart, Tasmania, Australia; 5 Health Services Innovation Tasmania, School of Medicine, Faculty of Health, University of Tasmania, Hobart, Tasmania, Australia; University of South Carolina School of Medicine, UNITED STATES

## Abstract

Inflammatory bowel diseases, such as ulcerative colitis, cause significant morbidity and decreased quality of life. The currently available treatments are not effective in all patients, can be expensive and have potential to cause severe side effects. This prompts the need for new treatment modalities. Enoxaparin, a widely used antithrombotic agent, is reported to possess anti-inflammatory properties and therefore we evaluated its therapeutic potential in a mouse model of colitis. Acute colitis was induced in male C57BL/6 mice by administration of dextran sulfate sodium (DSS). Mice were treated once daily with enoxaparin via oral or intraperitoneal administration and monitored for colitis activities. On termination (day 8), colons were collected for macroscopic evaluation and cytokine measurement, and processed for histology and immunohistochemistry. Oral but not intraperitoneal administration of enoxaparin significantly ameliorated DSS-induced colitis. Oral enoxaparin-treated mice retained their body weight and displayed less diarrhea and fecal blood loss compared to the untreated colitis group. Colon weight in enoxaparin-treated mice was significantly lower, indicating reduced inflammation and edema. Histological examination of untreated colitis mice showed a massive loss of crypt architecture and goblet cells, infiltration of immune cells and the presence of edema, while all aspects of this pathology were alleviated by oral enoxaparin. Reduced number of macrophages in the colon of oral enoxaparin-treated mice was accompanied by decreased levels of pro-inflammatory cytokines. Oral enoxaparin significantly reduces the inflammatory pathology associated with DSS-induced colitis in mice and could therefore represent a novel therapeutic option for the management of ulcerative colitis.

## Introduction

Ulcerative colitis (UC) is a chronic inflammatory condition with millions of sufferers worldwide [1]. In UC, chronic inflammation of the inner lining of the colon leads to abdominal pain, diarrhea, bloody stool and weight loss, and results in decreased quality of life. Currently, pharmacological and surgical interventions are the two main management approaches for UC [2]. Drugs such as corticosteroids, aminosalicylates, and immunosuppresants, which aim to decrease inflammation, show limited effectiveness for long term remission and are associated with significant side effects [3]. Monoclonal antibodies, such as infliximab, that inhibit tumour necrosis factor (TNF-α) have shown considerable success [4]. However, they are expensive, about 30–40% of patients do not respond to TNF-α inhibitors and in up to 40% of patients the initial therapeutic response is lost after 1–2 years [5]. Surgery is reserved for about 20–30% of patients who are unresponsive to medication and develop life-threatening complications such as perforation, refractory rectal bleeding, toxic megacolon and fulminant colitis [2]. Even after surgery, patients are predisposed to the risk of complications such as small bowel obstruction, anastomotic strictures, pouchitis and pouch failure [3].

Therefore, the search for safer and more effective agents for the management of UC continues. Among these agents, heparins (unfractionated heparin, UFH and low-molecular-weight heparins, LMWHs) have attracted much interest for their paradoxical responses. In clinical practice, heparins are widely used for the treatment and prophylaxis of venous thromboembolism [6]. However, many studies have demonstrated therapeutic usefulness of heparins apart from their anticoagulant activity, of which their anti-inflammatory properties have attracted much interest among researchers around the world [7].

Heparins are glycosaminoglycans and are composed of a heterogeneous mixture of highly sulphated anticoagulant and non-anticoagulant molecules known as oligosaccharides [8]. LMWHs have replaced UFH clinically to a large extent because of their improved pharmacokinetic properties and fewer side effects [6]. Thromboembolic complications have been reported in patients with UC and it was found serendipitously that UFH is linked to remission of UC when patients were treated with UFH for deep vein thrombosis or pulmonary embolism [9]. Since then, several pre-clinical as well as open-labelled clinical studies have demonstrated the usefulness of heparins in UC [10–14].

However, some pre-clinical and clinical studies have shown little or no significant clinical advantage of LMWHs compared to placebo when used for the management of UC [15–19]. The observed differences in clinical outcome could be due to the fact that these studies were not only heterogeneous in regards to their methodological approaches and definitions for clinical outcomes, but also used different heparins. For example, two LMWHs, dalteparin and nadroparin, have reported to be effective in patients with steroid resistant UC [14, 20]. On the other hand, tinzaparin (another LMWH) has shown no additive benefit over standard therapy in patients with UC [16]. LMWHs are prepared by different chemical or enzymatic processes and are dissimilar to each other in both their physical and chemical properties [8]. Many studies have shown real and significant differences between various LMWHs based on their oligosaccharide analysis [21, 22]. Dotan *et al*. found that enoxaparin may be an effective therapy for active UC and may delay or preclude the need for corticosteroid treatment [10]. On the other hand, Zezos and co-workers reported no additional therapeutic benefit of enoxaparin compared with classical therapy [17]. *In vivo* investigations of enoxaparin in other inflammatory conditions (e.g. lichen planus) have also reported contradictory results [23, 24]. Commercially available enoxaparin or any other LMWH is normally standardized according to its anticoagulant activity (anticoagulant oligosaccharides) but not the other molecules (non-anticoagulant oligosaccharides), which results in batch-to-batch variation [25, 26]. It has been reported that the differences in non-anticoagulant oligosaccharides between different batches of enoxaparin may be responsible for the inconsistent clinical findings [23]. We have also previously reported the structural variations in two different batches of enoxaparin formulated by the same manufacturer [26].

It is known that the absorption of LMWHs from the gut is poor after oral administration because of their hydrophilicity, high negative charge and large size [27]. Therefore, in previous clinical studies LMWHs were administered parenterally. However, such macromolecules are composed of anticoagulant and non-anticoagulant oligosaccharides and hence the risk of bleeding is increased when used for medical conditions other than where an anticoagulant effect is required. For example, in one study, administration of a LMWH resulted in the massive haemorrhage in a patient with UC [28]. Therefore, studies investigating the role of LMWHs in inflammatory conditions have used low parenteral doses in an attempt to avoid bleeding complications. However, it has been reported that their anti-inflammatory activities are expressed with high LMWH concentrations where the anticoagulant effects also predominate [29]. If heparins are administered orally, because of their lack of systemic absorption, they can be delivered to the site of inflammation in UC. This route would potentially minimise the risk of bleeding as well as allow the use of high doses. Given the mixed findings with the use of enoxaparin in UC, we investigated and compared the efficacy of orally and parenterally administered enoxaparin utilizing the most widely used animal model of chemically-induced colitis, with the aim of determining the probable underlying mechanisms of enoxaparin in attenuating colitis.

## Materials and Methods

### Animal colitis model

All animal experiments were approved by the Animal Ethics Committee of the University of Tasmania (Ethics approval number: A13576) and conducted in accordance with the Australian Code of Practice for Care and Use of Animals for Scientific Purposes (8^th^ Edition 2013). Male C57BL/6 mice, (aged 8–10 weeks; 21–30 g, average ≈ 25 g), were obtained from the University of Tasmania animal breeding facility and housed in a temperature-controlled environment with a 12-hour day/night light cycle. Individual body weights were assessed daily over an initial acclimation period of 7 days. All mice were non-fasting and had access to food and autoclaved tap water for drinking *ad libitum* during experiment. Colitis was induced by feeding mice with 3% w/v dextran sulfate sodium (DSS, MW = 40000–50000, USB, Affymetrix Inc, Ohio, USA) dissolved in drinking water from day 1 to day 8. Control mice received water without DSS from day 1 to day 8.

### Formulation of enoxaparin

Enoxaparin solution for intraperitoneal administration was prepared by diluting enoxaparin (100 mg/mL; Clexane, Sanofi Aventis, NSW, Australia) with water for injection to obtain a final concentration of 0.25 mg/mL. Food mash containing enoxaparin for oral administration was prepared by dissolving 9.6 g sucrose (4% w/w of food mash) in 100 mL autoclaved tap water followed by addition of 0.4 mL of enoxaparin (100 mg/mL) solution. Powdered food (130 g) (Barastoc Rat & Mouse Pellet, Ridley AgriProducts, Melbourne, Australia) was then added slowly with constant stirring to this solution to prepare a homogenous food mash mixture. Then 3 g of this mixture containing 0.5 mg of enoxaparin was transferred to food trays. Each food tray was stored at -20°C until used.

### Treatment with enoxaparin

Mice were assigned randomly into six groups: i) untreated healthy control mice (C, n = 6) ii) healthy mice treated with oral enoxaparin (C+OE, n = 3) iii) healthy mice treated with intraperitoneal enoxaparin (C+IPE, n = 3) iv) mice which received DSS (through drinking water) and vehicle (water for injection or food mash) (DSS, n = 14) v) mice which received DSS in drinking water and treated with oral enoxaparin (DSS+OE, n = 12) and vi) mice which received DSS in drinking water and were treated with intraperitoneal enoxaparin (DSS+IPE n = 6). Enoxaparin (20 mg/kg/day for oral or 0.5 mg/kg/day for intraperitoneal injection) was given from day 1 to day 7.

### Evaluation of intestinal inflammation

Mice were monitored daily for change in body weight. Stool consistency and the presence of blood in the stool were scored daily as described previously [30]. The anal area was examined for the presence of blood and feces was collected and tested for the presence of blood using Hemoccult II slides (Beckman Coulter Inc., California, USA).

### Termination of experiment and tissues sampling

Mice were killed by carbon dioxide inhalation followed by cervical dislocation on day 8. The entire colon was carefully removed and examined macroscopically. The colon length was measured before opening it longitudinally for observation of colonic content. After mechanical cleaning, colon weight was determined and the colon tissues were then divided longitudinally for histology and measurement of cytokine levels.

### Histologic evaluation of colitis

Colon tissue was fixed with 10% v/v buffered formalin and processed for paraffin embedding. Paraffin embedded tissue was cut into sections (4 μm thickness) before using for hematoxylin and eosin (H&E) staining and immunohistochemistry. All H&E sections were graded blindly for the severity of tissue damage at distal and proximal regions as described previously [31–33].

### Immunohistochemistry

For immunohistochemical staining, antigen retrieval was performed by incubating the sections for 10 minutes at 97°C in 1 mM EDTA buffer, pH 8 or 10 mM citrate buffer, pH 6. Activity of endogenous peroxidase was blocked by incubating sections with 3% v/v hydrogen peroxide for 20 minutes. Sections were then washed with 0.05 M Tris-buffered saline containing 0.5% v/v Tween 20 (TBST), pH 7.6. Subsequently, sections were incubated with serum-free protein block (Dako, Victoria, Australia) for 10 minutes. Colon sections were then incubated with primary antibodies: anti-F4/80 (ab111101, Abcam, Cambridge, UK, 1:100); anti-claudin-4 (ab15104, Abcam, 1:200); anti-occludin (ab64482, Abcam, 1:50) or its isotype control antibody (monoclonal rabbit immunoglobulin G, ab172730, Abcam, assay dependent concentration) overnight at 4°C or at room temperature for 1 hour. Sections were then washed 3 × 5 minutes and allowed to react with secondary antibody: anti-rabbit immunoglobulin G conjugated to horseradish peroxidase (HRP) (ab7090, Abcam, 1:300) at room temperature for 1 hour. Histological signal was developed using 3,3’-diaminobenzidine in a chromogen solution (ab64238, Abcam, 1:50) before counterstaining the sections with hematoxylin. Sections were dehydrated and mounted using mounting medium (Dako, Victoria, Australia) and then examined microscopically (Leica DM2500, Image Pro Plus 7.0 software) for positively stained cells. The number of F4/80-positive macrophages was counted at high-power field (400 × magnification) and averaged from ten fields per tissue section.

For double immunofluorescence staining, sections were dewaxed and rehydrated before antigen retrieval using 10 mM citrate buffer, pH 6 for 15 minutes at 97°C. Sections were incubated with serum-free protein block (Dako, Victoria, Australia) and permeabilized with 0.4% v/v Triton-X at room temperature for 30 minutes. Sections were incubated with primary antibodies: anti-F4/80 (ab16911, Abcam, 1:25); anti-IL1β (ab9722, Abcam, 1:300), anti-iNOS (ab136918, Abcam, 1:100), anti-mannose receptor (CD206) (ab64693, Abcam, 1:2000) overnight at 4°C or at room temperature for 1 hour. The omission of primary antibodies and replacement with isotype controls (rabbit immunoglobulin G and normal rat serum at assay-dependent concentration) served as independent negative controls. Sections were washed with TBST 3 × 10 minutes and incubated with species-specific secondary antibodies: anti-rat IgG H&L AlexaFluor 594 (ab150160, Abcam, 1:1000) and anti-rabbit IgG H&L AlexaFluor 488 (A11070, Thermo Fisher Scientific, Melbourne, Australia, 1:1000) at room temperature for 2 hours. Sections were rinsed with TBST 3 × 10 minutes, followed by a quick wash with distilled water before mounting using Glycerol Mounting Medium (Abcam) that contained 4’,6-diamidino-2-phenylindole (DAPI) and 1,4-diazobicyclo-2,2,2-octane (DABCO). Labelled tissues were visualized using a Leica DM LB2 microscope. Fluorescence images (400 × magnification) were captured using NIS-Elements 4.13 (Nikon) software. The number of fluorescence-positive cells was counted from five representative high-power fields (400 × magnification) per tissue section and expressed as percent of double positive cells/ total number of macrophages.

### Tissue explant culture and measurement of cytokine levels

Each collected tissue was cut, weighed and washed with cold PBS before transferring to a 12 well plate containing 1 mL / well of RPMI1640 culture medium (In Vitro technologies Pty Ltd, Melbourne, Australia) supplemented with 10% v/v fetal calf serum (Gibco, Life Technologies, Melbourne, Australia), penicillin (100 mU/L) and streptomycin (100 mg/L) (Sigma-Aldrich Pty Ltd, Sydney, Australia). After 24 hours of incubation, supernatant was collected from each well and stored at -80°C until further analysis. The cytokine levels were determined by immunoassay using a Bio-Plex Pro Mouse cytokine 23-plex kit (Bio-Rad Laboratories, Inc., Hercules, CA, USA) following manufacturer instructions. Briefly, standards were prepared by reconstituting the cytokine standard with culture media. Solution containing coupled magnetic beads was added into each well of a 96 well plate. Standard and samples were then added in duplicate and incubated for 30 minutes. After that, detection antibodies were added and incubated before final incubation with streptavidin-phycoerythrin. Cytokine levels were measured and analysed using a Bio-Plex 200 instrument (Bio-Rad Laboratories) and Bioplex Manager software, version 6 (Bio-Rad Laboratories) respectively. The cytokine levels were normalized by dividing the cytokine results (pg/mL) by the measured biopsy weight (mg) to obtain pg / mL of cytokines/ 10 mg of tissue.

### Statistical analysis

Results are presented as mean ± standard deviation (SD), or points of minimum, median, mean, maximum and interquartile range. Using GraphPad Prism (version 6, GraphPad Software Inc, CA, USA), statistical significance was evaluated using one or two way analysis of variance (ANOVA) followed by multiple comparison test: Dunnett’s test to evaluate the difference between each treatment group and the colitis control group or Tukey’s test to determine the differences between different groups. Pearson’s correlation coefficient (r^*2*^) was determined for the relationship between two variables when necessary. A *p* value of < 0.05 was considered statistically significant.

## Results

### Orally administered enoxaparin ameliorates colitis-induced weight loss

**Fig 1:**
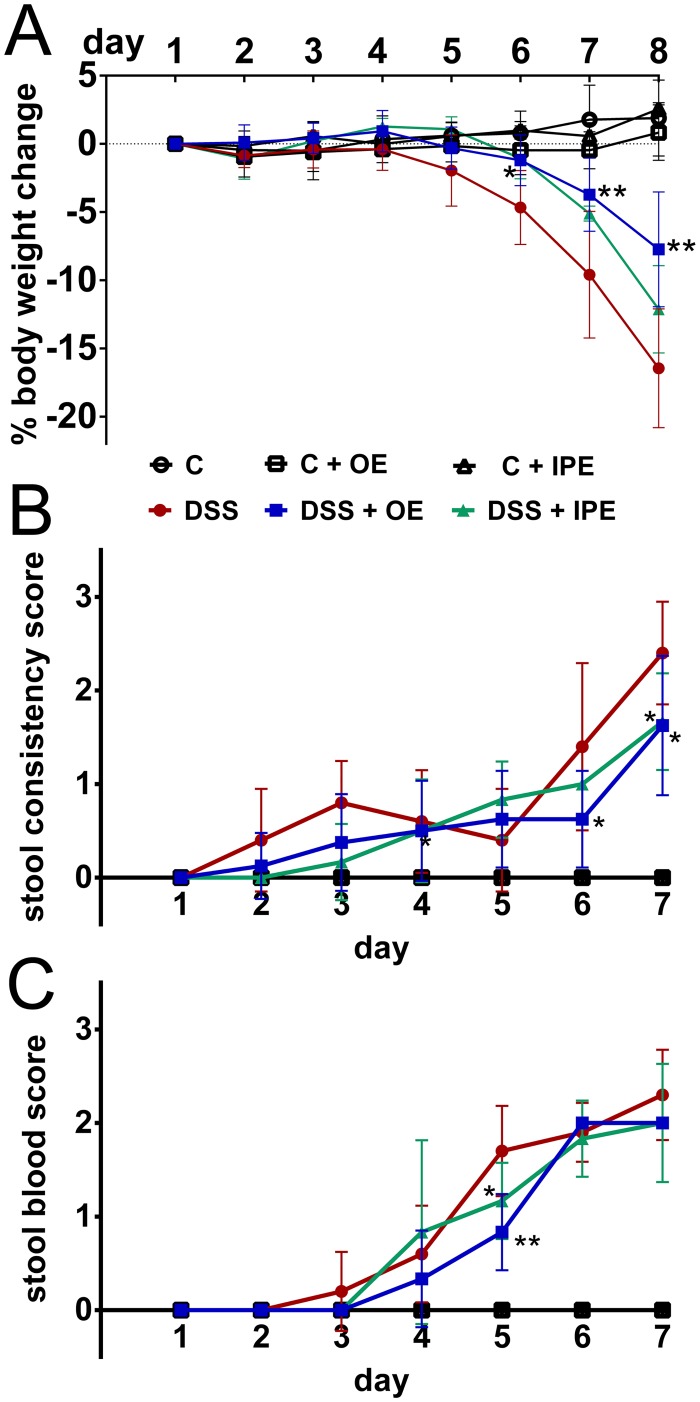
Effect of enoxaparin during acute colitis. (A) Daily body weight changes during colitis induction in C57BL/6 mice with and without enoxaparin compared to healthy control. Stool samples collected from mice were scored for (B) consistency and the presence of (C) occult blood on a daily basis during experiment. Results are expressed as mean ± SD of n = 3–14 mice. * *p* < 0.05 and ** *p* < 0.01. Control, C; control with oral enoxaparin, C+OE; control with intaperitoneal injection of enoxaparin, C+IPE; untreated colitis, DSS; colitis with oral enoxaparin, DSS+OE; colitis with intraperitoneal injection of enoxaparin, DSS+IPE.

In line with previous reports [34], we observed a reduction of approximately 15% body weight over an 8 day DSS treatment period (Fig 1A). Oral enoxaparin attenuated body weight loss where the difference in the body weight loss between DSS-treated (DSS) and oral enoxaparin treated (DSS+OE) mice was significant on day 6 (2.9%, *p* = 0.0007), day 7 (4.9%, *p* < 0.0001) and day 8 (8.1%, *p* < 0.0001) (Fig 1A). On the other hand, difference in the body weight loss between DSS and intraperitoneal enoxaparin (DSS+IPE) was not significant from day 1 to day 8 (Fig 1A).

### Enoxaparin ameliorates colitis-associated diarrhea and blood in stool

Over the treatment period, we observed an increased occurrence of loose stool and diarrhea in mice receiving DSS (Fig 1B). Consistent with an amelioration of weight loss, orally administered enoxaparin significantly reduced the occurrence of loose stool or diarrhea on day 6 and day 7 (Fig 1B). On the other hand, intraperitoneal enoxaparin also reduced the mean stool consistency score on day 7 (Fig 1B). Both oral and intraperitoneal enoxaparin significantly prevented the presence of blood in stool on day 5 only; this effect was not seen on day 6 and 7 (Fig 1C). There was no statistical difference between oral and intraperitoneal enoxaparin treatment for both mean stool consistency and stool blood scores.

### Orally administered enoxaparin alleviates macroscopic changes of the colon

**Fig 2:**
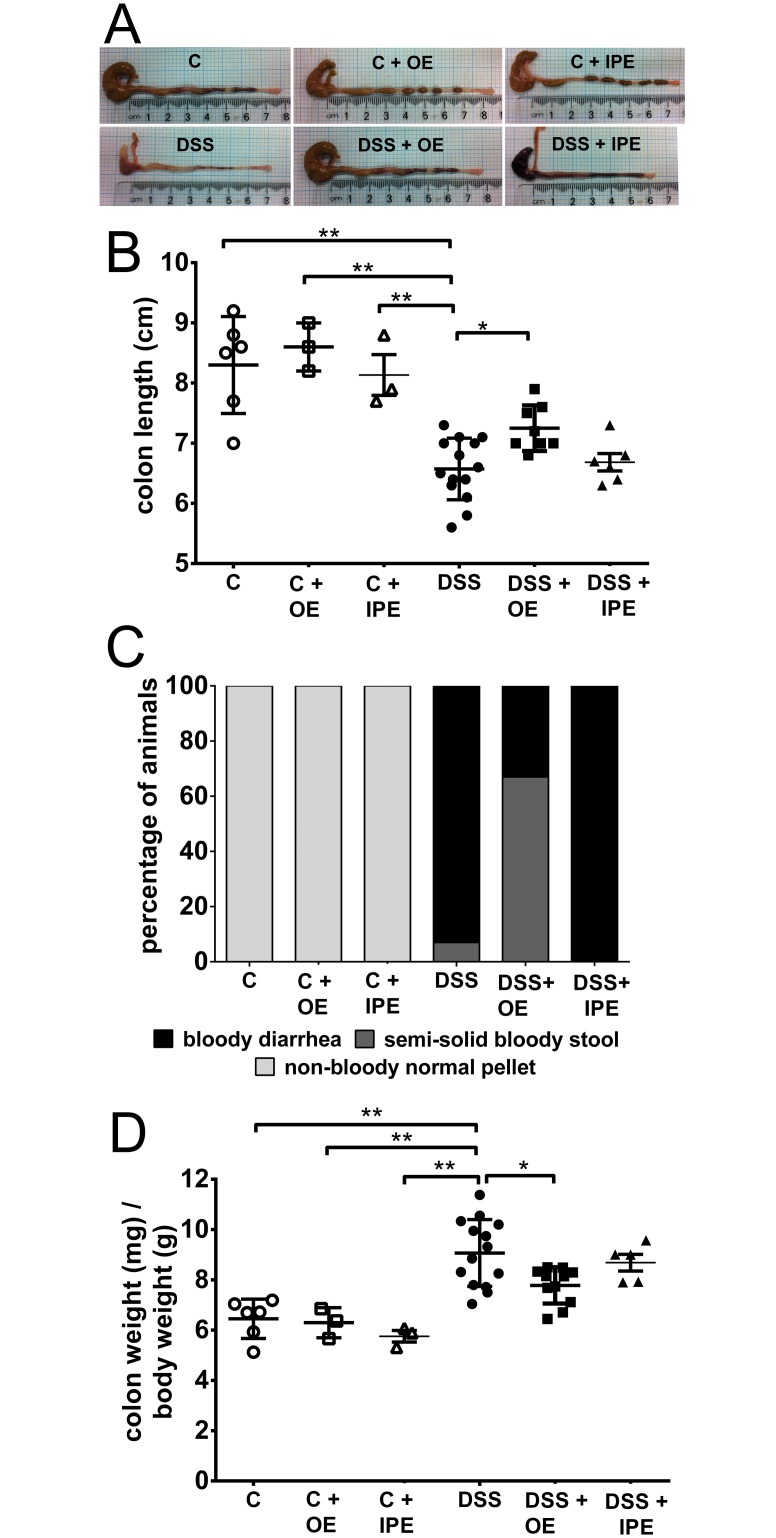
Effect of enoxaparin on macroscopic appearance of colon. (A) Representative images of colons from mice treated with and without enoxaparin. (B) The colons were measured for their length. (C) The appearance of colon luminal content was evaluated before the measurement of colon weight. (D) The relative colon weight was presented as colon weight divided by body weight. Results are expressed as mean ± SD of n = 3–14 mice. * *p* < 0.05 and ** *p* < 0.01. Control, C; control with oral enoxaparin, C+OE; control with intraperitoneal injection of enoxaparin, C+IPE; untreated colitis, DSS; colitis with oral enoxaparin, DSS+OE; colitis with intraperitoneal injection of enoxaparin, DSS+IPE.

Having observed beneficial clinical changes by orally administered enoxaparin, we then investigated the effects of enoxaparin in ameliorating macroscopic changes of colon (Fig 2A & S1 Fig). Acute colitis was associated with a significant shortening of the colon. The length of colon was reduced by 20.8% compared to healthy control (Fig 2B). Oral enoxaparin significantly prevented shortening of colon by 10.2% (*p* = 0.025) compared to DSS. Intraperitoneal enoxaparin had no effect on the length of colon (Fig 2B). Oral enoxaparin also reduced the severity of gross changes in the luminal contents of the distal colon compared to untreated colitis (Fig 2C).

Colon weight change, a well-known independent marker of intestinal edema and inflammation, was presented as colon weight over body weight. Oral enoxaparin suppressed the increase in relative colon weight by 14.2% (*p* = 0.011) compared to untreated colitis. On the other hand, intraperitoneal enoxaparin reduced the relative colon weight by only 4.2%, which was not significant (*p* = 0.91) (Fig 2D).

### Orally administered enoxaparin decreases colon damage and infiltration of inflammatory cells

**Fig 3:**
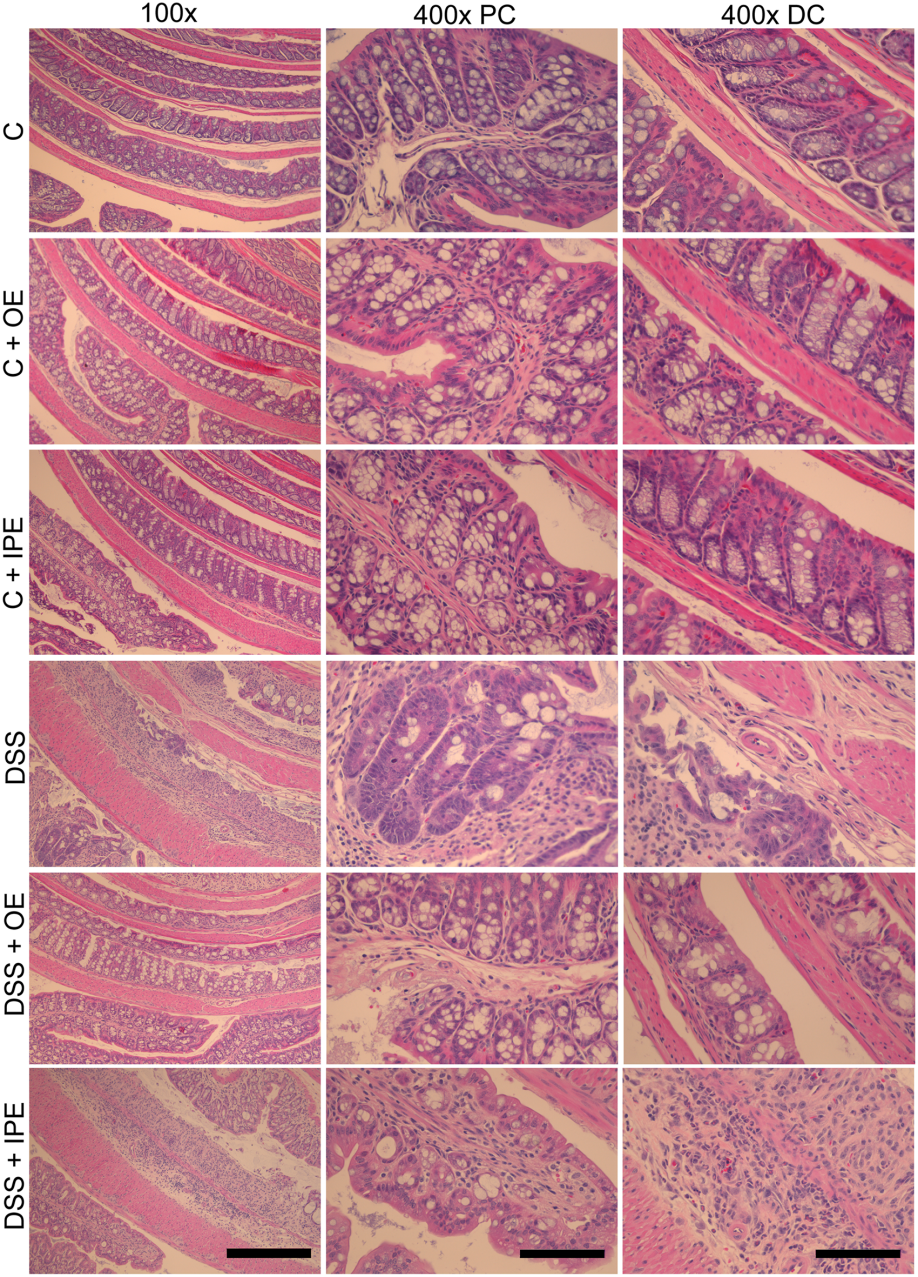
Effect of enoxaparin on histological changes of colon. Representative hematoxylin and eosin stained colon sections of healthy and colitis mice with and without enoxaparin. Scale bars = 100 μm for 400 × and 400 μm for 100 × magnification. Control, C; control with oral enoxaparin, C+OE; control with intraperitoneal injection of enoxaparin, C+IPE; untreated colitis, DSS; colitis with oral enoxaparin, DSS+OE; colitis with intraperitoneal injection of enoxaparin, DSS+IPE.

**Fig 4:**
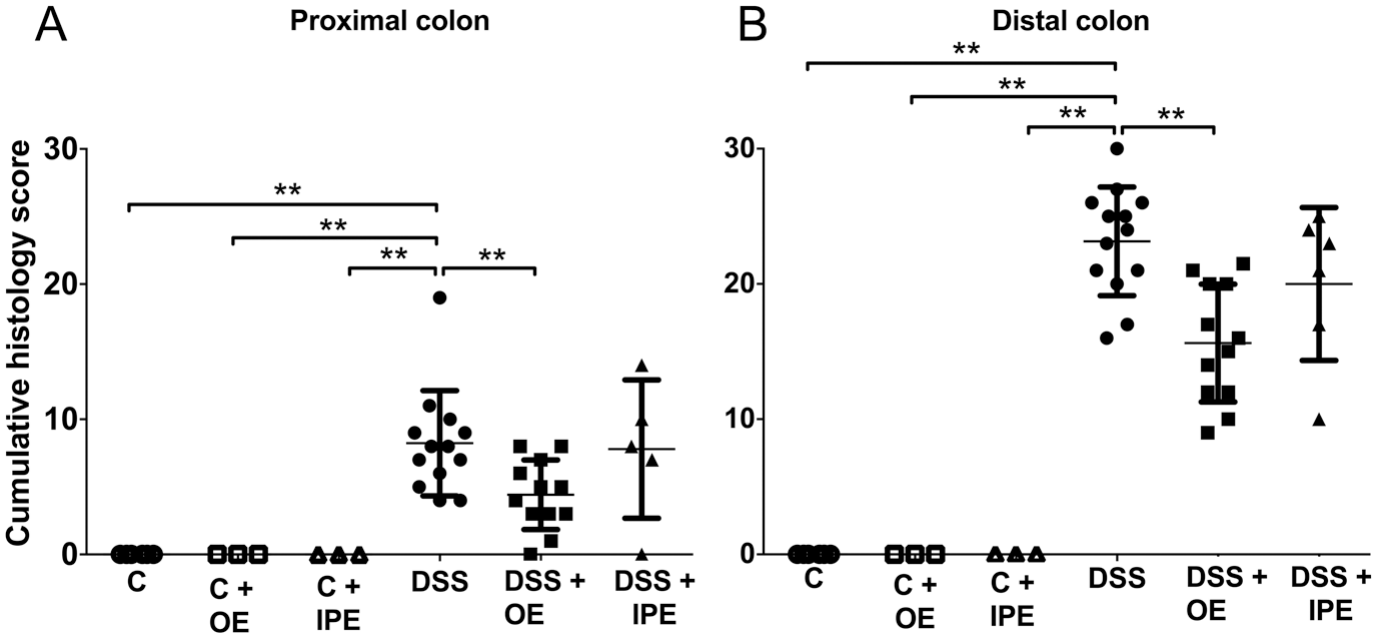
Effect of enoxaparin on histological changes of colon. Cumulative histology damage scores for (A) proximal colon and (B) distal colon. Results are expressed as mean ± SD of n = 3–14 mice. ** *p* < 0.01. Control, C; control with oral enoxaparin, C+OE; control with intraperitoneal injection of enoxaparin, C+IPE; untreated colitis, DSS; colitis with oral enoxaparin, DSS+OE; colitis with intraperitoneal injection of enoxaparin, DSS+IPE.

Colons of healthy untreated mice showed intact surface epithelium, intact mucosa and submucosa, non-disrupted crypt architecture, complete goblet cells with mucus vacuoles and only a small number of leukocytes (Fig 3A). In contrast, DSS treated mice showed structural damage and infiltration of inflammatory cells into the colon. Careful evaluation of colon tissues revealed regional DSS-induced injuries mainly confined to the distal colon where diffused destruction of crypt architecture, goblet cell loss, submucosa edema and increased infiltration of inflammatory cells was evident (Fig 3A). Oral enoxaparin reduced the disruption of crypt architecture and epithelium, reduced globlet cell loss, showed less severe submucosa edema, and also reduced infiltration of inflammatory cells (Fig 3). The cumulative histological scores for both the proximal colon (Fig 4A) and distal colon (Fig 4B) were significantly lower in orally treated mice with colitis compared to untreated colitis (4 versus 8 and 16 versus 23 respectively). Intraperitoneal administration of enoxaparin did not reduce the colon damage and infiltration of inflammatory cells (Fig 3). Also, the cumulative histological scores were not statistically different compared to DSS (Fig 4).

### Orally administered enoxaparin reduces the levels of pro-inflammatory cytokines

**Fig 5:**
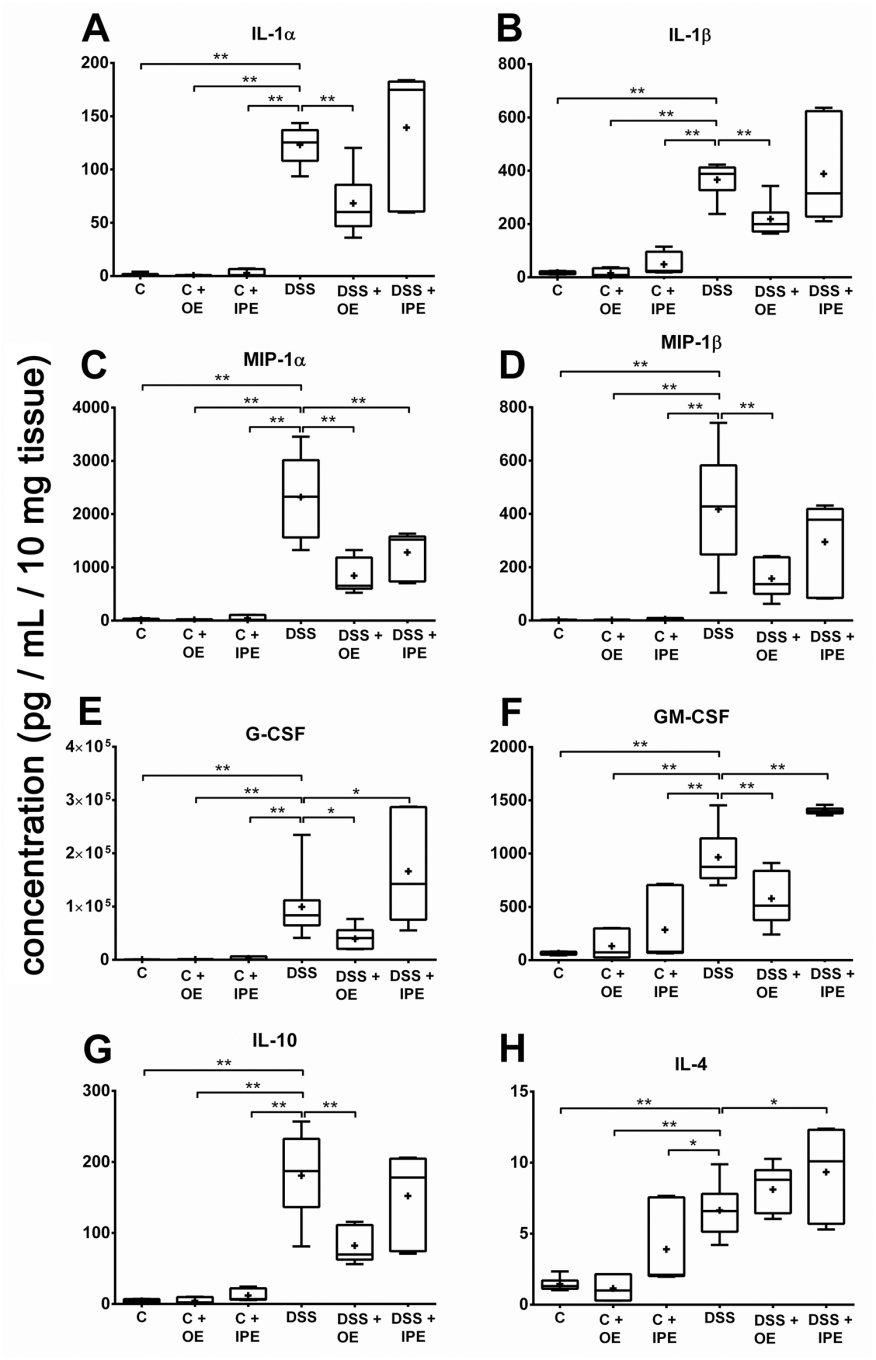
Effect of enoxaparin on colonic cytokine levels. Distal colon tissues of mice were cultured for 24 hours. Supernatants were collected and cytokine levels were measured using Bio-Plex assay. Cytokine levels in the supernatant were normalized to tissue weight to obtain pg / mL of cytokines/ 10 mg of tissue. Results are expressed as minimum, 25th percentile, median, mean, 75th percentile and maximum of cytokine levels of 3–5 mice. * *p* < 0.05 and ** *p* < 0.01. Interleukin, IL; macrophage inflammatory protein, MIP; granulocyte colony-stimulating factor, G-CSF; granulocyte–macrophage colony-stimulating factor, GM-CSF; Control, C; control with oral enoxaparin, C+OE; control with intraperitoneal injection of enoxaparin, C+IPE; untreated colitis, DSS; colitis with oral enoxaparin, DSS+OE; colitis with intraperitoneal injection of enoxaparin, DSS+IPE.

As expected, colon tissues of mice administered with DSS showed significant increase in a wide range of inflammatory cytokines (Fig 5 & S2 Fig). Oral enoxaparin reduced the levels of a number of cytokines significantly. The levels of IL-1α, IL-1β, IL-10, MIP-1α, MIP-1β, G-CSF and GM-CSF were reduced by 44.6% (*p* = 0.0002), 40.4% (*p* = 0.0011), 54.5% (*p* < 0.0001), 63.6% (*p* = 0.0002), 62.3% (*p* = 0.0263), 51.6% (*p* = 0.0013) and 40.1% (*p* < 0.0001) respectively (Fig 5A–5G). However intraperitoneal enoxaparin produced inconsistent response. For example, it reduced MIP-1α level by approximately 45% (Fig 5C). On the other hand, the levels of G-CSF, GM-CSF and IL-4 were increased significantly (Fig 5E, 5F, and 5H). Unlike intraperitoneal enoxaparin, oral enoxaparin did not affect the levels of cytokines in healthy mice.

**Fig 6:**
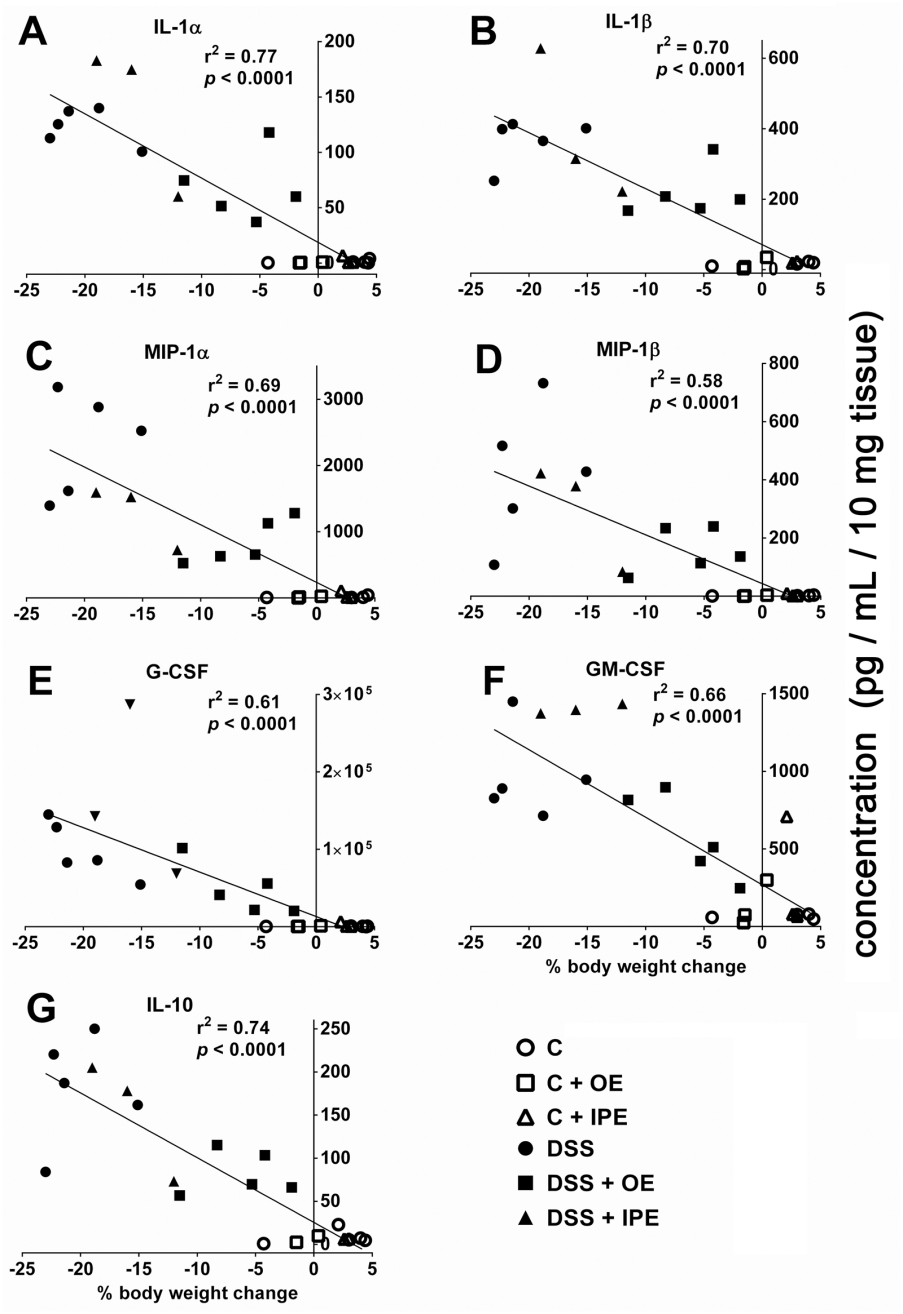
Correlation between colonic cytokine levels and body weight changes during acute colitis. Changes in cytokine levels were correlated with the changes of body weight of individual mice. The value of the Pearson correlation coefficient (r^2^) is reported and significance is indicated by *p* value. Interleukin, IL; macrophage inflammatory protein, MIP; granulocyte colony-stimulating factor, G-CSF; granulocyte–macrophage colony-stimulating factor, GM-CSF; Control, C; control with oral enoxaparin, C+OE; control with intraperitoneal injection of enoxaparin, C+IPE; untreated colitis, DSS; colitis with oral enoxaparin, DSS+OE; colitis with intraperitoneal injection of enoxaparin, DSS+IPE.

Since increased cytokine levels in the colonic mucosa of UC patients are closely related to the severity of inflammation and tissue damage [35–37], we determined the Pearson’s correlation coefficients between cytokine levels (that statistically significantly decreased by oral treatment) and the percentage body weight change in individual mice on day 8. Consistent with clinical observations, cytokine levels significantly and positively correlated with the severity of body weight loss (Fig 6A–6G). Mucosal concentrations of IL-1α (r^2^ = 0.77, *p* < 0.0001), IL- 1β (r^2^ = 0.70, *p* < 0.0001), MIP-1α (r^2^ = 0.69, *p* < 0.0001), MIP-1β (r^2^ = 0.58, *p* < 0.0001), G-CSF (r^2^ = 0.61, *p* < 0.0001), GM-CSF (r^2^ = 0.66, *p* < 0.0001), IL-10 (r^2^ = 0.74, *p* < 0.0001) were each substantially correlated with the percentage of body weight loss.

### Orally administered enoxaparin affects macrophage numbers and differentiation status during colitis

**Fig 7:**
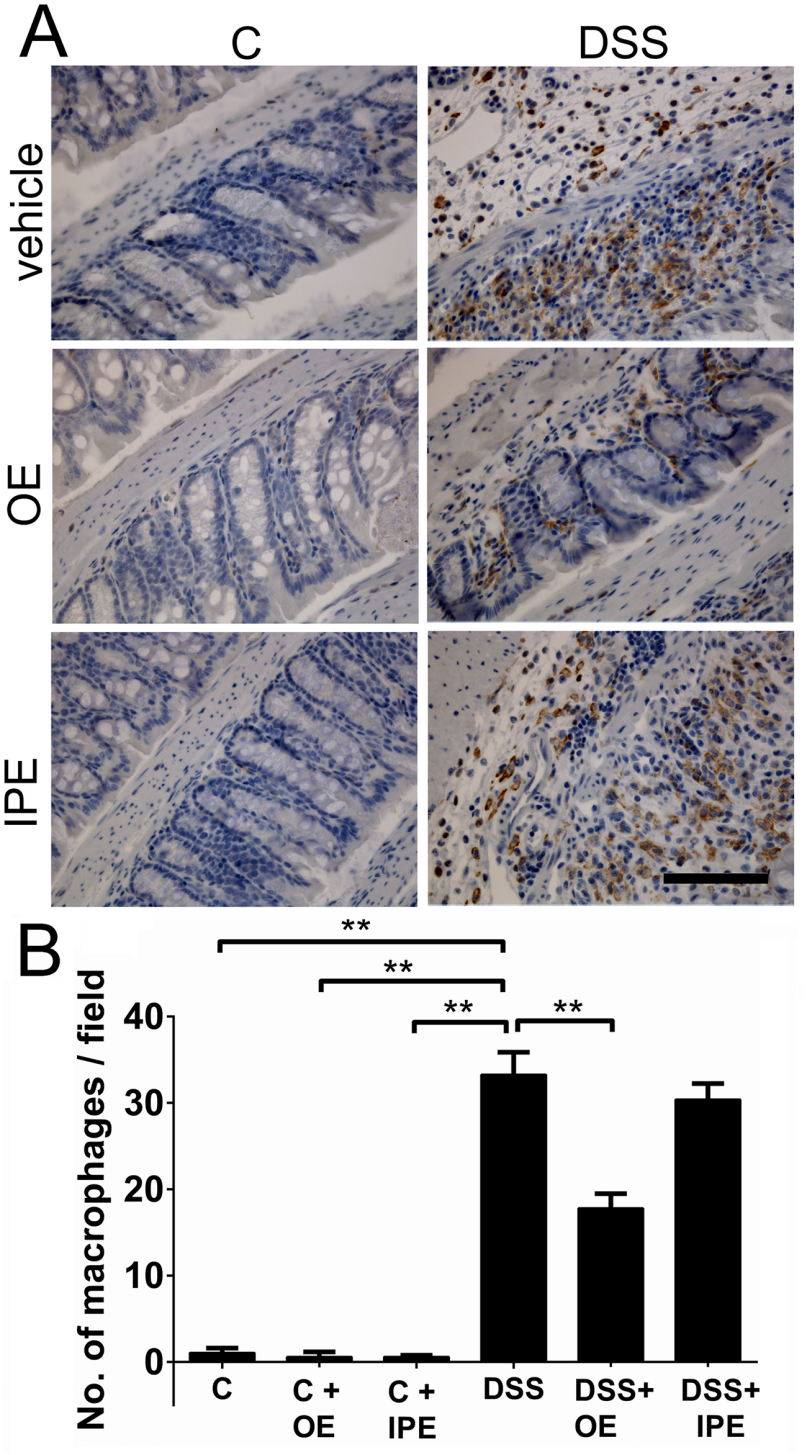
Effect of enoxaparin on macrophage infiltration into the inflamed colon. (A) Representative immunostaining of F4/80-positive macrophages in in the distal colon from healthy and colitic mice treated with and without enoxaparin. (B) Quantification of macrophages (F4/80^+^ cells) in the colons. Results are expressed as mean ± SD of ten representative high-power fields per tissue section of 3–5 mice each. ** *p* < 0.01. Scale bar = 100 μm for 400 × magnification. Control, C; untreated colitis, DSS; oral enoxaparin, OE; intraperitoneal injection of enoxaparin, IPE.

Macrophages play an important role during acute colitis by secreting various pro-inflammatory mediators [38]. Therefore, we investigated if the observed reduction in cytokines could be a result of reduced number of macrophages in the colonic mucosa. When we compared the number of macrophages between different types of treatment, significantly higher macrophage numbers (33.3 ± 7.1/ field) were observed in the mucosa and submucosa in DSS-treated mice compared to healthy control mice (1.1 ± 1.2/ field) (Fig 7). Consistent with the reduced cytokine levels, oral enoxaparin also significantly decreased the number of macrophages (17.8 ± 5.4/ field, *p* < 0.0001) in the mucosa and submucosa. On the other hand, intraperitoneal enoxaparin was not effective in decreasing the number of macrophages numbers in the colon tissue (29.2 ± 6.5/ field) (Fig 7).

**Fig 8:**
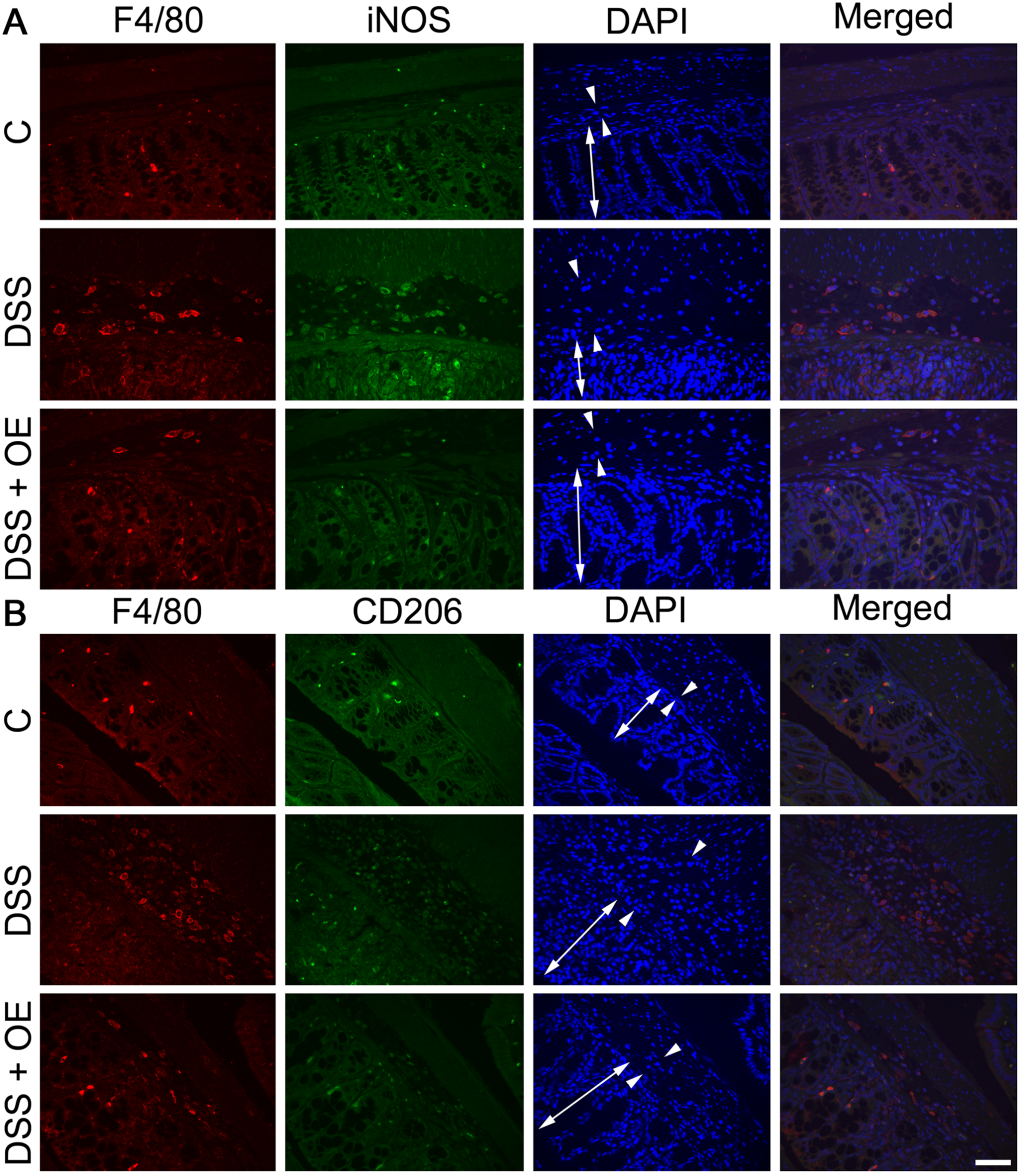
Effect of enoxaparin on macrophage phenotypes. Co-immunostaining of macrophages and their phenotypes. Representative images of (A) M1 macrophages (F4/80^+^ and iNOS^+^) and (B) M2 macrophages (F4/80^+^ and CD206^+^) using colon tissue from n = 3–5 mice. F4/80 positive cells were visualized using Alexa Fluor 594-conjugated goat anti-rat IgG (red) and iNOS or CD206 positive cells using Alexa Fluor 488-conjugated goat anti-rabbit IgG (green). Nuclei were stained with 4’,6-diamidino-2-phenylindole (DAPI, blue). Localization of mucosa (double-headed arrow) and submucosa (arrowheads) is indicated. Scale bar = 50 μm for 400 × magnification. Control, C; untreated colitis, DSS; colitis with oral enoxaparin, DSS+OE.

In addition to the absolute numbers of macrophages, we also looked at their differentiation status. When we detected M1 macrophages (F4/80 and iNOS positive cells, Fig 8A) in the colon tissue, only low numbers were observed mainly in the mucosa (double headed arrow) of healthy control colons. In response to DSS treatment, as expected large numbers of M1 macrophages were detected not only within the edematous submucosa (arrow heads) but also within the mucosa (double headed arrow). In response to enoxaparin treatment, those macrophage numbers were significantly reduced with a few M1 cells present only in the mucosa (double headed arrow) (Fig 8A).

In contrast, when we looked at M2 macrophages (F4/80 and CD-206 positive cells, Fig 8B), the majority of macrophages were present in the mucosa of healthy colon (double headed arrow). In response to DSS, only a very low numbers of M2 macrophages were present in the mucosa and submucosa, while in response to enoxaparin treatment, larger numbers of M2 macrophages were present in the mucosa and submucosa (Fig 8B).

**Fig 9:**
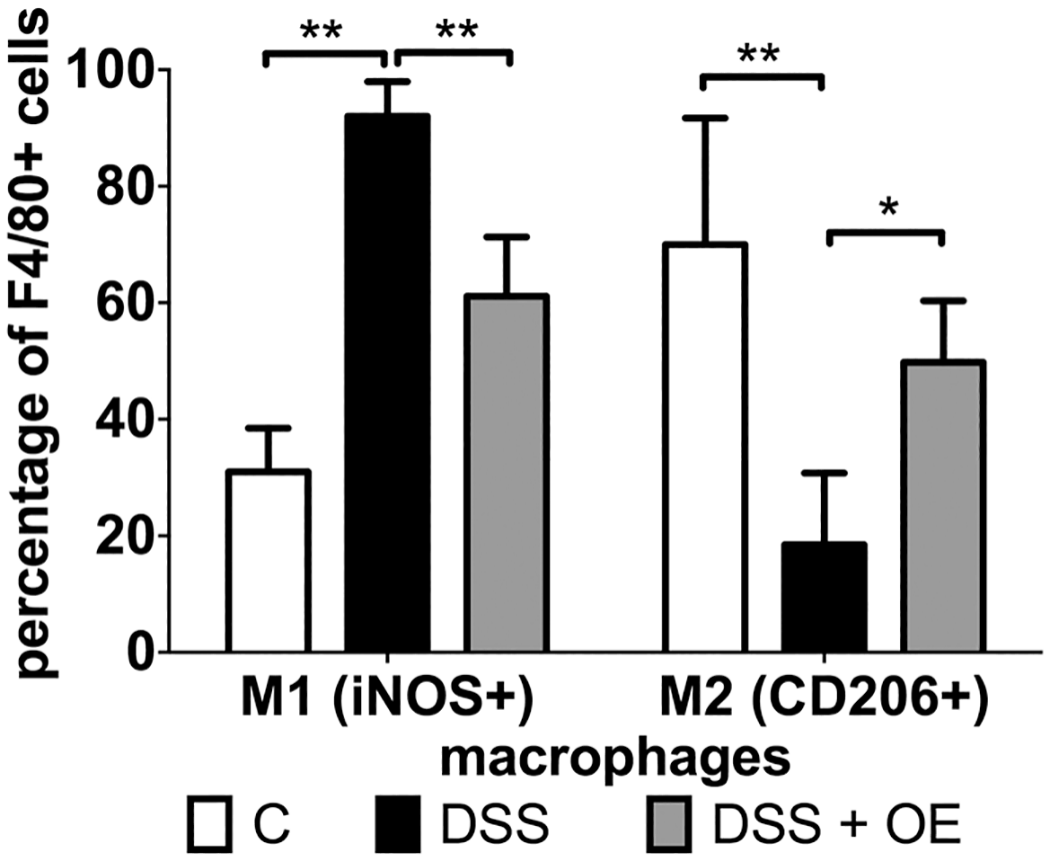
Effect of enoxaparin on macrophage phenotypes. Quantification of macrophages expressing M1 and M2 phenotype markers in the colon. Results are expressed as percentage of double-positive macrophages from total macrophages ± SD of five representative high-power fields per tissue section of n = 3–5 mice each. * *p* < 0.05 and ** *p* < 0.01.

Quantitative analysis of these histological results showed that overall, a healthy colon contained twice as many M2 compared to M1 macrophages (open bars, Fig 9). In contrast, the M1/M2 ratio increased markedly in DSS-induced colitis, with 92.0 ± 5.9% of macrophages being M1 while only a minority were M2 cells (black bars, Fig 9). On the other hand, oral enoxaparin treatment significantly increased the levels of M2 macrophages by 31.3% (p = 0.03), whereas the M1 cells were reduced by 30.9% (p = 0.0007) compared to untreated colitis colons (grey bars, Fig 9).

**Fig 10:**
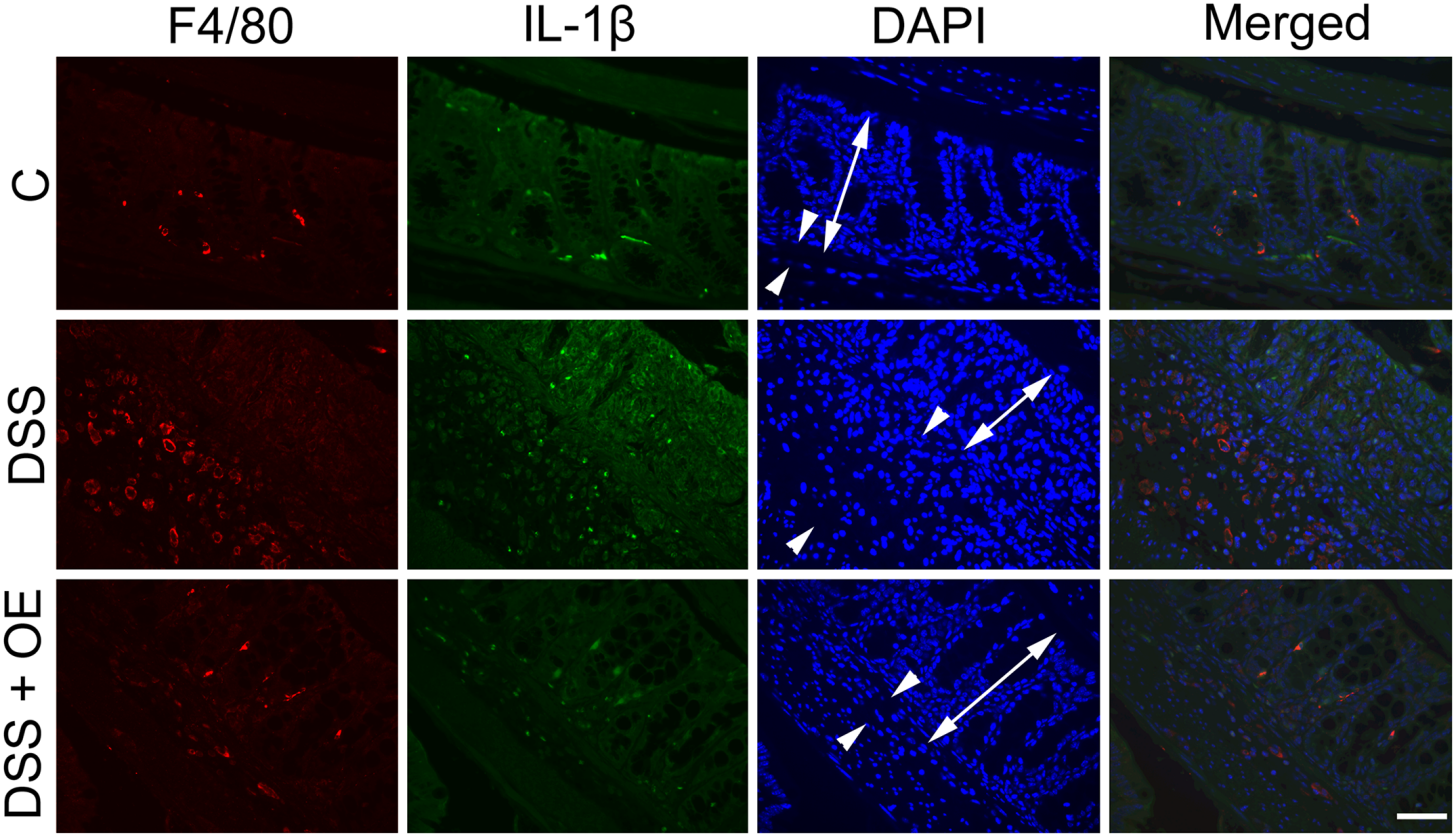
Effect of enoxaparin on IL-1β expression on colon tissues. Co-immunostaining of macrophages and IL-1β. Representative images of IL-1β and F4/80 staining of colons from n = 3–5 mice. F4/80 positive cells were visualized using Alexa Fluor 594-conjugated goat anti-rat IgG (red) and IL-1β positive cells using Alexa Fluor 488-conjugated goat anti-rabbit IgG (green). Nuclei were stained with 4’,6-diamidino-2-phenylindole (DAPI, blue). Localization of mucosa (double-headed arrow) and submucosa (arrowheads) is indicated. Scale bar = 50 μm for 400 × magnification. Control, C; untreated colitis, DSS; colitis with oral enoxaparin, DSS+OE.

IL-1β acts as central mediator of pro-inflammatory immune responses. We therefore investigated a possible mechanistic link between reduced IL-1β-levels and reduced M1 macrophage numbers in enoxaparin-treated mice (Fig 10). Relative to healthy mice, IL-1β immune-labelling increased under conditions of untreated colitis. At the same time, in the enoxaparin-treated colon, we observed a reduced expression of IL-1β confirming our previous results (Fig 5). However, using co-localization of IL-1β staining with detection of a macrophage marker (F4/80), we were unable to demonstrate that IL-1β was expressed by macrophages in this disease model (Fig 10).

### Orally administered enoxaparin retains occludin and claudin-4 by attenuating crypt loss

**Fig 11:**
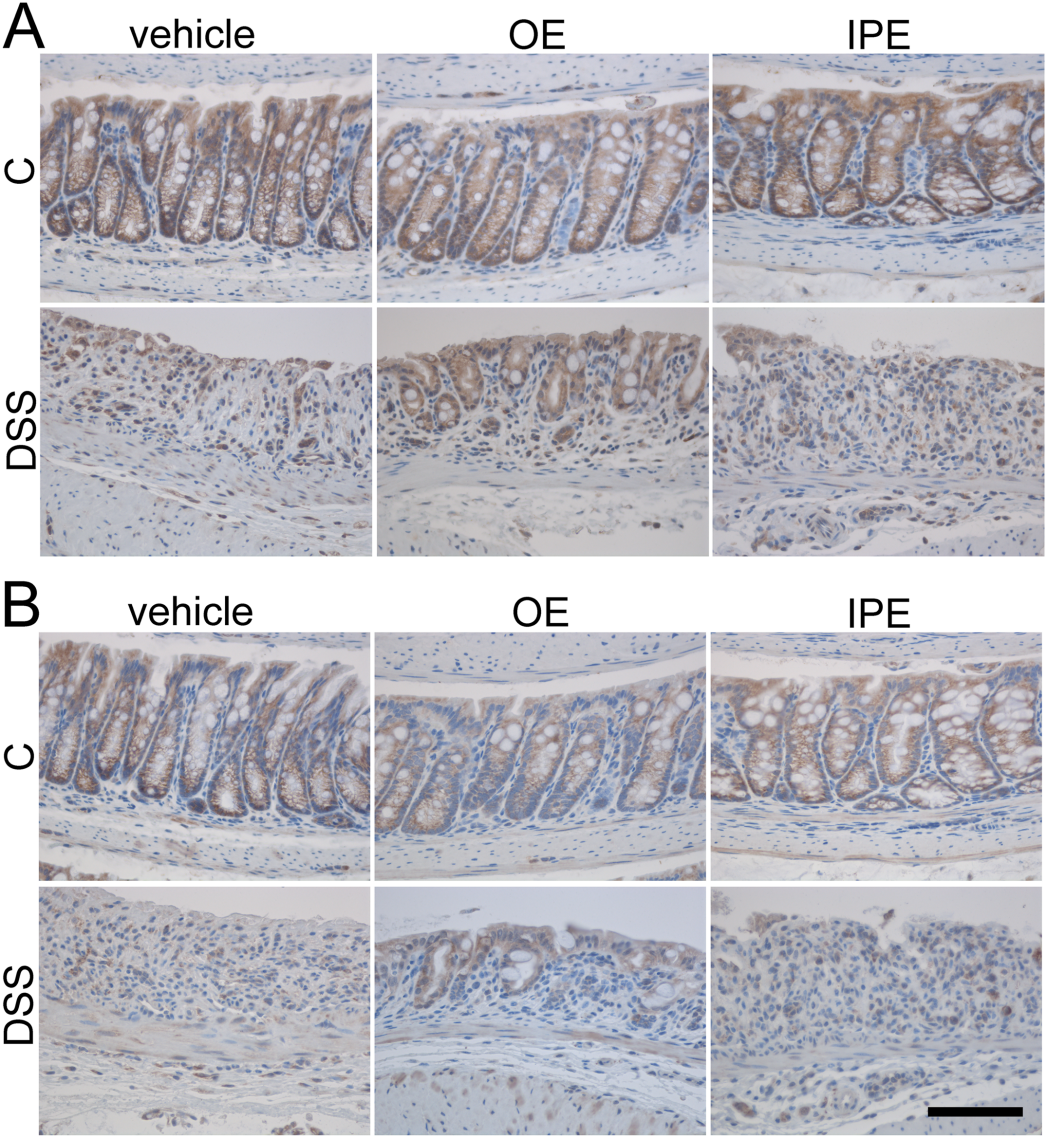
Effect of enoxaparin on epithelial tight junctions of colon. Immunostaining of (A) occludin and (B) claudin-4 in the distal colon of healthy and colitis mice treated with and without enoxaparin. Scale bar = 100 μm for 400 × magnification. Control, C; untreated colitis, DSS; oral enoxaparin, OE; intraperitoneal injection of enoxaparin, IPE.

The intact intestinal epithelium serves as a protective layer, which is maintained by the intercellular interactions between tight junction proteins such as occludin and claudins [39]. Immunohistochemistry was performed to determine the localization of tight junction proteins, occludin and claudin-4. As shown in Fig 11, occludin and claudin-4 proteins were homogenous as well as continuous on colonic epithelial cell membrane in healthy control. In contrast, in sections with untreated colitis, massive mucosal erosion and crypt loss prevented the reliable detection of tight junction proteins (Fig 11). However, in mice receiving oral enoxaparin, colonic occludin and claudin-4 staining indicated a greater proportion of intact colonic crypts (Fig 11). On the other hand, intraperitoneal enoxaparin resulted in a tight junction protein staining comparable to untreated colitis mice, while enoxaparin treatment in healthy mice via the diet or by intraperitoneal injection did not alter the distribution or quantity of tight junction proteins in the colon.

## Discussion

In this study, we have described the therapeutic potential of the low molecular weight heparin, enoxaparin, for the amelioration of acute colitis. Oral enoxaparin reduced the severity of clinical activity, histological damage and the immunological response associated with colitis. To our knowledge, this is the first report to demonstrate that enoxaparin given through the diet is highly effective in ameliorating experimental colitis, while inducing no mortality and adverse effects in healthy mice. Consistent with other study [40], our results showed that enoxaparin given as intraperitoneal injection did not reduce the severity of colitis. To date, animal studies investigating the efficacy of different doses of subcutaneously administered enoxaparin have reported inconsistent results. For example, subcutaneous enoxaparin at 80 μg/kg/day over 7 days effectively ameliorated experimental colitis, while this effect was not observed at lower (40 μg/kg/day) or higher (200 μg/kg/day) doses in the same study [11]. Likewise, positive effects for subcutaneous enoxaparin at doses of 250–280 μg/kg/day have been reported [41, 42], while enoxaparin at 3 mg/kg/day was shown to be less efficacious [43]. Overall, our results are comparable to previous studies showing that enoxaparin is more effective when administered orally and not parenterally. Oral enoxaparin was previously reported to reduce the severity of colitis [43, 44], while on the other hand intraperitoneal enoxaparin at a dose of ten times higher (5 mg/kg/day) than our study (0.5 mg/kg/day) also did not relieve colitis-associated symptoms in mice [40], in agreement with our results. One limitation of prior studies is the uncertainty around the local availability of drug in the intestine when delivered subcutaneously or intraperitoneally, which is further complicated by observations that subcutaneous administration of enoxaparin produced inconsistent results at different doses as describe above. At present it is unclear if the reported discrepancies are a result of different dosing regimens or if they are related to the use of different pre-clinical colitis models. Overall, our results, together with the reports described above, strongly suggest that after parenteral administration, LMWHs cannot reliably reach areas of inflammation at the intestinal mucosa or submucosa. In contrast, current evidence supports the idea that oral administration is effective to deliver enoxaparin locally. From a patient’s perspective, this is not only the preferred option, but this route of administration is likely to restrict the systemic availability of LMWHs, which therefore reduces the risk of bleeding complications.

Previously, the disease activity index was commonly used to summarize three clinical scores of colitis [42, 43]. In this study however, we evaluated individual clinical parameters on a daily basis to identify differences of colitis severity and to better understand treatment-specific responses. Using this approach, oral enoxaparin showed a significant protection against weight loss, diarrhea and intestinal bleeding as well as attenuation of histological features of colitis.

A multitude of cytokines is implicated in the pathogenesis of UC [45]. These mediators form a complex network that regulates mucosal inflammation and affect the integrity of epithelium [45]. Since pro-inflammatory cytokines from patient samples correlate with disease activity [35, 46], treatments that modulate these mediators are likely to be of therapeutic use. In our study, we quantified a larger number of cytokines compared to previous studies to get a broader understanding of the immunological cytokine response during intestinal inflammation and especially in response to enoxaparin treatment. The presence of elevated pro-inflammatory cytokines during intestinal inflammation was in general agreement with previous studies [38, 47, 48]. Although these studies reported on a significantly lower number of cytokines, overall they mirrored our results of a broad pro-inflammatory environment [47, 48]. Also, in our study, oral enoxaparin reduced the levels of a number of mucosal cytokines including IL-1α, IL-1β, IL-10, MIP-1α, MIP-1β, G-CSF, GM-CSF during colitis. The importance of individual cytokine levels is reflected by their good correlations with the colitis-induced body weight loss of each animal.

Macrophages found in the colon of active UC patients are key mediators of human UC [49] and induce tissue damage by secreting inflammatory cytokines in the colonic mucosa during acute colitis [38]. Discrete macrophage subsets with divergent effects are often grouped functionally as M1 (monocyte-derived or classically activated or pro-inflammatory) and M2 (tissue-resident or alternatively activated or anti-inflammatory) macrophages [49–51]. During intestinal inflammation, monocytes are recruited and differentiated into pro-inflammatory macrophages within the lamina propria under the influence of GM-CSF [49]. This growth factor is necessary for the development, differentiation and proliferation of monocyte-derived inflammatory macrophages and is known to contribute to M1 polarization [51]. As expected by the reduced levels of GM-CSF in response to oral enoxaparin treatment, reduced numbers of M1 macrophages and elevated numbers of M2 macrophages were detected. These results are in agreement with other reports where M1 macrophages contributed critically to DSS-induced colitis, while M2 macrophages were protective [52, 53]. Although our data suggest that that oral enoxaparin effectively reduced GM-CSF level, which could in part be responsible for suppressed M1/M2 ratio, this observation has to be confirmed in detailed future studies. Since macrophages are a major source of other pro-inflammatory cytokines, this connection could also explain the reduction of multiple other cytokines such as IL-1α, IL-1β, IL-10, MIP-1α and MIP-1β that originated from inflamed colon tissues. However, the precise source of cytokines in this model of colitis is not completely known at present and these mediators could in principle be secreted by a variety of cells during inflammation. In human inflammatory bowel disease, it is thought that IL-1β is expressed by macrophages in the inflamed colon [54]. In contrast, in our mouse model, we were unable to demonstrate IL-1β expression in intestinal macrophages. Together with the strong reduction of IL-1β in colon tissue in response to enoxaparin, this suggests that enoxaparin either does not only target macrophages or that the observed changes to IL-1β level are a secondary effect. In contrast to previous observations [11, 44], the expression of TNF-α was not significantly affected by enoxaparin treatment. TNF-α is thought to play significant role in inflammatory cellular signalling which is reflected by the successful clinical use of TNF-α inhibitors in UC patients [4]. However, it is likely that a number of factors including disease location, type of inflammation, pathogenic mechanisms and levels of multiple cytokines in combination affect the response to colitis treatment [45]. Therefore, agents that target a single pro-inflammatory cytokine are likely limited in their ability to offer an effective maintenance therapy over extended periods of time, while simultaneously modulating multiple pro-inflammatory mediators to reduce maturation and infiltration of immune cells could provide a more sustainable strategy against inflammation.

Epithelial barrier integrity is essential to a healthy gut function [55] and our understanding of how epithelial homeostasis is altered in response to intestinal inflammation is indispensable to develop therapeutic interventions that facilitate mucosal healing and normalize epithelial functions. In UC, the reduction of tight junctions, associated with increased in intestinal permeability and impaired epithelial function [56–58] is at least partially caused by chronic inflammation [55]. In line with our results, previous studies reported a disrupted and irregular expression pattern of tight junction proteins including occludin and claudin-4 in the colonic mucosa in mice with DSS-induced colitis [59, 60]. However, in this study, DSS-treatment led to massive epithelial damage with loss of epithelial cells that prevented us to reliably quantify tight junction proteins. Nevertheless, both tight junction proteins could act as surrogate markers to indicate the effect of oral treatment as it correlates with the retention of crypt architecture. Since it is likely that oral enoxaparin is mainly acting locally to relieve the severity of colitis [43], it is conceivable that its interaction with epithelial cells could directly protect them against DSS-induced damage. Protecting epithelial integrity would retain the epithelial barrier function and protect against infiltration of microbial antigens into lamina propria. The retention of crypt architecture could be the main reason for the localized and regular staining of tight junction proteins in colitis mice treated with oral enoxaparin, which is similar to the staining observed in healthy mice.

Although heparins are well-known for their non-anticoagulant effects [7], their exact mode of action in UC remain unclear. Different mechanisms including a reduced infiltration of leukocytes as well as pro-inflammatory mediators have been postulated [11, 61, 62]. Heparins are structurally similar to heparan sulphate. Loss of cell surface heparan sulphate proteoglycans (HSPG) is reported in patients with UC, resulting in decreased intestinal mucosal healing [63]. It has been also postulated that heparins can increase mucosal healing in UC or during colitis by substituting the loss of cell surface HSPG [40, 63]. Like other LMWHs and UFH, enoxaparin is also composed of a complex mixture of structurally unknown oligosaccharides. Further work to identify the specific components of enoxaparin that are responsible for the observed effects will be a pre-requisite to identify their mode(s) of action as well as to progress the most promising molecules towards clinical trials.

Overall, enoxaparin given at an early stage of colitis significantly prevents the development of colitis and reduces the pathology associated with acute colitis induced by DSS. This study extends our current understanding of oral administration of enoxaparin during acute colitis, which is a crucial step towards the use of enoxaparin for the treatment of colitis. The ability of enoxaparin to reduce inflammation and retain epithelial integrity along with the possibility of oral delivery to provide a better safety profile for clinical use, serves as a rationale to develop enoxaparin components as a therapeutic alternative for patients with UC.

## Supporting Information

S1 Fig Representative images of gross appearance of cecum. C57BL/6 mice were given 3% w/v DSS in their drinking water from day 1 to day 8. Control mice were given water only. They were treated with or without oral (p.o.) enoxaparin. Cecums were collected on day of termination. Control, C; control with oral enoxaparin, C+OE; untreated colitis, DSS; colitis with oral enoxaparin, DSS+OE.
(TIF)

S2 Fig Effects of enoxaparin on colonic cytokine levels. Distal colon tissues of mice were cultured for 24 hours. Supernatants were collected and measured for cytokine levels by using Bio-Plex assay. Cytokine levels in the supernatant were normalized to tissue weight to obtain pg / mL of cytokines/ 10 mg of tissue. Results are expressed as minimum, 25th percentile, median, mean, 75th percentile and maximum of cytokine levels of 3–5 mice. * *p* < 0.05 and ** *p* < 0.01. Out of range, OOR; Interleukin, IL; interferon, IFN; keratinocyte-derived chemokine, KC; monocyte chemotactic protein-1, MCP-1; regulated and normal T cells expressed and secreted, RANTES; tumor necrosis factor-α, TNF-α; Control, C; control with oral enoxaparin, C+OE; control with intraperitoneal injection of enoxaparin, C+IPE; untreated colitis, DSS; colitis with oral enoxaparin, DSS+OE; colitis with intraperitoneal injection of enoxaparin, DSS+IPE.
(TIF)

## References

[1] Cosnes J, Gower-Rousseau C, Seksik P, Cortot A. Epidemiology and natural history of inflammatory bowel diseases. Gastroenterology. 2011;140: 1785–1794. 10.1053/j.gastro.2011.01.055 21530745

[2] Ordás I, Eckmann L, Talamini M, Baumgart DC, Sandborn WJ. Ulcerative colitis. Lancet. 2012;380: 1606–1619. 10.1016/S0140-6736(12)60150-0 22914296

[3] Triantafillidis JK, Merikas E, Georgopoulos F. Current and emerging drugs for the treatment of inflammatory bowel disease. Drug Des Devel Ther. 2011;5: 185–210. 10.2147/DDDT.S11290 21552489 PMC3084301

[4] Sands BE, Kaplan GG. The role of TNFα in ulcerative colitis. J Clin Pharmacol. 2007;47: 930–941. 17567930 10.1177/0091270007301623

[5] Ferrante M, Vermeire S, Fidder H, Schnitzler F, Noman M, Van Assche G, et al Long-term outcome after infliximab for refractory ulcerative colitis. J Crohns Colitis. 2008;2: 219–225. 10.1016/j.crohns.2008.03.004 21172214

[6] Hirsh J, Warkentin TE, Shaughnessy SG, Anand SS, Halperin JL, Raschke R, et al Heparin and low-molecular-weight heparin mechanisms of action, pharmacokinetics, dosing, monitoring, efficacy, and safety. Chest. 2001;119: 64S–94S. 11157643 10.1378/chest.119.1_suppl.64s

[7] Lever R, Page C. Non-anticoagulant effects of heparin: an overview In: Lever R, Mulloy B, Page CP, editors. Heparin—A Century of Progress. Handbook of Experimental Pharmacology. 207: Heidelberg, Berlin: Springer; 2012 p. 281–305. 10.1007/978-3-642-23056-1_12 22566229

[8] Linhardt RJ, Gunay NS. Production and chemical processing of low molecular weight heparins. Semin Thromb Hemost. 1999;25: 5–16. 10549711

[9] Gaffney PR, Doyle CT, Gaffney A, Hogan J, Hayes D, Annis P. Paradoxical response to heparin in 10 patients with ulcerative colitis. Am J Gastroenterol. 1995;90: 220–223. 7847289

[10] Dotan I, Hallak A, Arber N, Santo M, Alexandrowitz A, Knaani Y, et al Low-dose low-molecular weight heparin (enoxaparin) is effective as adjuvant treatment in active ulcerative colitis: an open trial. Dig Dis Sci. 2001;46: 2239–2244. 11680603 10.1023/a:1011979418914

[11] Dotan I, Hershkoviz R, Karmeli F, Brazowski E, Peled Y, Rachmilewitz D, et al Heparin and low-molecular-weight heparin (enoxaparin) significantly ameliorate experimental colitis in rats. Aliment Pharmacol Ther. 2001;15: 1687–1697. 11564011 10.1046/j.1365-2036.2001.01079.x

[12] Panés J, Estevet M, Cabré E, Hinojosa J, Andreu M, Sans M, et al Comparison of heparin and steroids in the treatment of moderate and severe ulcerative colitis. Gastroenterology. 2000;119: 903–908. 11040177 10.1053/gast.2000.18159

[13] Pastorelli L, Saibeni S, Spina L, Signorelli C, Celasco G, De Franchis R, et al Oral, colonic-release low-molecular-weight heparin: an initial open study of Parnaparin-MMX for the treatment of mild-to-moderate left-sided ulcerative colitis. Aliment Pharmacol Ther. 2008;28: 581–588. 10.1111/j.1365-2036.2008.03757.x 18700898

[14] Vrij A, Jansen J, Schoon E, Brüine AD, Hemker H, Stockbrügger R. Low molecular weight heparin treatment in steroid refractory ulcerative colitis: clinical outcome and influence on mucosal capillary thrombi. Scand J Gastroenterol. 2001;36: 41–47. 10.1080/003655201753265091 11768560

[15] Korzenik J, Hsu A, Robert M. Effect of heparin on dextran sulfate sodium-induced colitis. Dig Dis Sci. 1998;43: 1800–1805. 9724172 10.1023/a:1018800207063

[16] Bloom S, Kiilerich S, Lassen MR, Forbes A, Leiper K, Langholz E, et al Low molecular weight heparin (tinzaparin) vs. placebo in the treatment of mild to moderately active ulcerative colitis. Aliment Pharmacol Ther. 2004;19: 871–878. 15080848 10.1111/j.1365-2036.2004.01926.x

[17] Zezos P, Papaioannou G, Nikolaidis N, Patsiaoura K, Papageorgiou A, Vassiliadis T, et al Low molecular weight heparin (enoxaparin) as adjuvant therapy in the treatment of active ulcerative colitis: a randomized, controlled, comparative study. Aliment Pharmacol Ther. 2006;23: 1443–1453. 16669959 10.1111/j.1365-2036.2006.02870.x

[18] de Bievre M, Vrij A, Schoon E, Dijkstra G, de Jong A, Oberndorff-Klein Woolthuis A, et al Randomized, placebo-controlled trial of low molecular weight heparin in active ulcerative colitis. Inflamm Bowel Dis. 2007;13: 753–758. 17260365 10.1002/ibd.20085

[19] Queiroz KC, Van 't Veer C, Van Den Berg Y, Duitman J, Versteeg HH, Aberson HL, et al Tissue factor-dependent chemokine production aggravates experimental colitis. Mol Med. 2011;17: 1119–1126. 10.2119/molmed.2011.00138 21717035 PMC3188874

[20] Törkvist L, Thorlacius H, Sjöqvist U, Bohman L, Lapidus A, Flood L, et al Low molecular weight heparin as adjuvant therapy in active ulcerative colitis. Aliment Pharmacol Ther. 1999;13: 1323–1328. 10540047 10.1046/j.1365-2036.1999.00599.x

[21] Bisio A, Vecchietti D, Citterio L, Guerrini M, Raman R, Bertini S, et al Structural features of low-molecular-weight heparins affecting their affinity to antithrombin. Thromb Haemost. 2009;102: 865–873. 10.1160/TH09-02-0081 19888521

[22] Ye H, Toby TK, Sommers CD, Ghasriani H, Trehy ML, Ye W, et al Characterization of currently marketed heparin products: Key tests for LMWH quality assurance. J Pharm Biomed Anal. 2013;85: 99–107. 10.1016/j.jpba.2013.06.033 23917037

[23] Hodak E, Yosipovitch G, David M, Ingber A, Chorev L, Lider O, et al Low-dose low-molecular-weight heparin (enoxaparin) is beneficial in lichen planus: a preliminary report. J Am Acad Dermatol. 1998;38: 564–568. 9555795 10.1016/s0190-9622(98)70118-5

[24] Rai R, Kaur I, Kumar B. Low-dose low-molecular-weight heparin in lichen planus. J Am Acad Dermatol. 2002;46: 141–143. 10.1067/mjd.2002.117389 11756963

[25] Fareed J, Hoppensteadt D, Schultz C, Ma Q, Kujawski MF, Messmore H. Biochemical and pharmacologic heterogeneity in low molecular weight heparins. Impact on the therapeutic profile. Curr Pharm Des. 2004;10: 983–999. 15078128 10.2174/1381612043452811

[26] Patel RP, Narkowicz C, Hutchinson JP, Hilder EF, Jacobson GA. A simple capillary electrophoresis method for the rapid separation and determination of intact low molecular weight and unfractionated heparins. J Pharm Biomed Anal. 2008;46: 30–35. 18024047 10.1016/j.jpba.2007.10.009

[27] Paliwal R, Paliwal SR, Agrawal GP, Vyas SP. Recent advances in search of oral heparin therapeutics. Med Res Rev. 2012;32: 388–409. 10.1002/med.20217 21287569

[28] Kaya S, Hatemi I, Hamzaoglu I, Uzunismail H. Massive haemorrhage induced by low molecular weight heparin in a patient with steroid refractory ulcerative colitis. Scand J Gastroenterol. 2004;39: 613–613. 15223691 10.1080/00365520410004622

[29] Celasco G, Papa A, Jones R, Moro L, Bozzella R, Surace M, et al Clinical trial: oral colon-release parnaparin sodium tablets (CB-01-05 MMX) for active left-sided ulcerative colitis. Aliment Pharmacol Ther. 2010;31: 375–386. 10.1111/j.1365-2036.2009.04194.x 19891665

[30] Wirtz S, Neufert C, Weigmann B, Neurath MF. Chemically induced mouse models of intestinal inflammation. Nat Protocols. 2007;2: 541–546. 17406617 10.1038/nprot.2007.41

[31] Eri R, McGuckin M, Wadley R. T cell transfer model of colitis: a great tool to assess the contribution of T cells in chronic intestinal inflammation In: Ashman RB, editor. Leucocytes: Methods and Protocols. Methods in Molecular Biology. 844: New York, NY: Humana Press; 2012 p. 261–275. 10.1007/978-1-61779-527-5_19 22262449

[32] Zheng B, van Bergenhenegouwen J, Overbeek S, van de Kant HJ, Garssen J, Folkerts G, et al *Bifidobacterium breve* attenuates murine dextran sodium sulfate-induced colitis and increases regulatory T cell responses. PLoS One. 2014;9: e95441 10.1371/journal.pone.0095441 24787575 PMC4008378

[33] Wlodarska M, Thaiss CA, Nowarski R, Henao-Mejia J, Zhang J-P, Brown EM, et al NLRP6 inflammasome orchestrates the colonic host-microbial interface by regulating goblet cell mucus secretion. Cell. 2014;156: 1045–1059. 10.1016/j.cell.2014.01.026 24581500 PMC4017640

[34] Rose W, Sakamoto K, Leifer C. Multifunctional role of dextran sulfate sodium for in vivo modeling of intestinal diseases. BMC Immunol. 2012;13: 41 10.1186/1471-2172-13-41 22853702 PMC3488029

[35] Banks C, Bateman A, Payne R, Johnson P, Sheron N. Chemokine expression in IBD. Mucosal chemokine expression is unselectively increased in both ulcerative colitis and Crohn's disease. J Pathol. 2003;199: 28–35. 12474223 10.1002/path.1245

[36] Yang S-K, Choi M-S, Kim O-H, Myung SJ, Jung H-Y, Hong W-S, et al The increased expression of an array of C-X-C and C-C chemokines in the colonic mucosa of patients with ulcerative colitis: regulation by corticosteroids. Am J Gastroenterol. 2002;97: 126–132. 11808935 10.1111/j.1572-0241.2002.05431.x

[37] Reimund JM, Wittersheim C, Dumont S, Muller C, Baumann R, Poindron P, et al Mucosal inflammatory cytokine production by intestinal biopsies in patients with ulcerative colitis and Crohn's disease. J Clin Immunol. 1996;16: 144–150. 8734357 10.1007/BF01540912

[38] Hall LJ, Faivre E, Quinlan A, Shanahan F, Nally K, Melgar S. Induction and activation of adaptive immune populations during acute and chronic phases of a murine model of experimental colitis. Dig Dis Sci. 2011;56: 79–89. 10.1007/s10620-010-1240-3 20467900

[39] Suzuki T. Regulation of intestinal epithelial permeability by tight junctions. Cell Mol Life Sci. 2013;70: 631–659. 10.1007/s00018-012-1070-x 22782113 PMC11113843

[40] Floer M, Götte M, Wild MK, Heidemann J, Gassar ES, Domschke W, et al Enoxaparin improves the course of dextran sodium sulfate-induced colitis in syndecan-1-deficient mice. Am J Pathol. 2010;176: 146–157. 10.2353/ajpath.2010.080639 20008145 PMC2797877

[41] Wang X, Chen Y, Song Y, Zhang S, Xie X. Activated syndecan-1 shedding contributes to mice colitis induced by dextran sulfate sodium. Dig Dis Sci. 2011;56: 1047–1056. 10.1007/s10620-010-1398-8 20936359

[42] Wang X-f, Li A-m, Li J, Lin S-y, Chen C-d, Zhou Y-l, et al Low molecular weight heparin relieves experimental colitis in mice by downregulating IL-1β and inhibiting syndecan-1 shedding in the intestinal mucosa. PLoS One. 2013;8: e66397 10.1371/journal.pone.0066397 23874391 PMC3715511

[43] Pellequer Y, Meissner Y, Ubrich N, Lamprecht A. Epithelial heparin delivery via microspheres mitigates experimental colitis in mice. J Pharmacol Exp Ther. 2007;321: 726–733. 17322027 10.1124/jpet.106.117226

[44] Luo J-Y, Zhong Y, Cao J-C, Cui H-F. Efficacy of oral colon-specific delivery capsule of low-molecular-weight heparin on ulcerative colitis. Biomed Pharmacother. 2011;65: 111–117. 10.1016/j.biopha.2010.12.013 21227626

[45] Neurath MF. Cytokines in inflammatory bowel disease. Nat Rev Immunol. 2014;14: 329–342. 10.1038/nri3661 24751956

[46] Ishiguro Y. Mucosal proinflammatory cytokine production correlates with endoscopic activity of ulcerative colitis. J Gastroenterol. 1999;34: 66–74. 10204613 10.1007/s005350050218

[47] Melgar S, Karlsson A, Michaëlsson E. Acute colitis induced by dextran sulfate sodium progresses to chronicity in C57BL/6 but not in BALB/c mice: correlation between symptoms and inflammation. Am J Physiol Gastrointest Liver Physiol. 2005;288: G1328–G1338. 15637179 10.1152/ajpgi.00467.2004

[48] Kim JJ, Shajib MS, Manocha MM, Khan WI. Investigating intestinal inflammation in DSS-induced model of IBD. J Vis Exp. 2012;60: e3678. 10.3791/3678 PMC3369627 22331082

[49] Heinsbroek SEM, Gordon S. The role of macrophages in inflammatory bowel diseases. Expert Rev Mol Med. 2009;11: e11. 19439108 10.1017/S1462399409001069

[50] Steinbach EC, Plevy SE. The role of macrophages and dendritic cells in the initiation of inflammation in IBD. Inflamm Bowel Dis. 2014;20: 166–175. 10.1097/MIB.0b013e3182a69dca 23974993 PMC4098861

[51] Italiani P, Boraschi D. From Monocytes to M1/M2 Macrophages: Phenotypical vs. Functional Differentiation. Front Immunol. 2014;5: 514 10.3389/fimmu.2014.00514 25368618 PMC4201108

[52] Zhu W, Yu J, Nie Y, Shi X, Liu Y, Li F, et al Disequilibrium of M1 and M2 macrophages correlates with the development of experimental inflammatory bowel diseases. Immunol Invest. 2014;43: 638–652. 10.3109/08820139.2014.909456 24921428

[53] Weisser SB, Brugger HK, Voglmaier NS, McLarren KW, van Rooijen N, Sly LM. SHIP-deficient, alternatively activated macrophages protect mice during DSS-induced colitis. J Leukoc Biol. 2011;90: 483–492. 10.1189/jlb.0311124 21685246

[54] McAlindon ME, Hawkey CJ, Mahida YR. Expression of interleukin 1 beta and interleukin 1 beta converting enzyme by intestinal macrophages in health and inflammatory bowel disease. Gut. 1998;42: 214–219. 9536946 10.1136/gut.42.2.214 PMC1726995

[55] Antoni L, Nuding S, Wehkamp J, Stange EF. Intestinal barrier in inflammatory bowel disease. World J Gastroenterol. 2014;20: 1165–1179. 10.3748/wjg.v20.i5.1165 24574793 PMC3921501

[56] Oshima T, Miwa H, Joh T. Changes in the expression of claudins in active ulcerative colitis. J Gastroenterol Hepatol. 2008;23: S146–S150. 10.1111/j.1440-1746.2008.05405.x 19120888

[57] Kucharzik T, Walsh SV, Chen J, Parkos CA, Nusrat A. Neutrophil transmigration in inflammatory bowel disease is associated with differential expression of epithelial intercellular junction proteins. Am J Pathol. 2001;159: 2001–2009. 11733350 10.1016/S0002-9440(10)63051-9 PMC1850599

[58] Schmitz H, Barmeyer C, Fromm M, Runkel N, Foss H-D, Bentze CJ, et al Altered tight junction structure contributes to the impaired epithelial barrier function in ulcerative colitis. Gastroenterology. 1999;116: 301–309. 9922310 10.1016/s0016-5085(99)70126-5

[59] Hayashi Y, Aoyagi K, Morita I, Yamamoto C, Sakisaka S. Oral administration of mesalazine protects against mucosal injury and permeation in dextran sulfate sodium-induced colitis in rats. Scand J Gastroenterol. 2009;44: 1323–1331. 10.3109/00365520903262414 19891583

[60] Bento AF, Marcon R, Dutra RC, Claudino RF, Cola M, Pereira Leite DF, et al β-Caryophyllene inhibits dextran sulfate sodium-induced colitis in mice through CB2 receptor activation and PPARγ pathway. Am J Pathol. 2011;178: 1153–1166. 10.1016/j.ajpath.2010.11.052 21356367 PMC3070571

[61] Belmiro CLR, Castelo-Branco MTL, Melim LMC, Schanaider A, Elia C, Madi K, et al Unfractionated heparin and new heparin analogues from ascidians (chordate-tunicate) ameliorate colitis in rats. J Biol Chem. 2009;284: 11267–11278. 10.1074/jbc.M807211200 19258310 PMC2670131

[62] Lever R, Smailbegovic A, Page CP. Locally available heparin modulates inflammatory cell recruitment in a manner independent of anticoagulant activity. Eur J Pharmacol. 2010;630: 137–144. 10.1016/j.ejphar.2009.12.015 20043903

[63] Day R, Ilyas M, Daszak P, Talbot I, Forbes A. Expression of syndecan-1 in inflammatory bowel disease and a possible mechanism of heparin therapy. Dig Dis Sci. 1999;44: 2508–2515. 10630505 10.1023/a:1026647308089

