# Supplementary figures and images for: Orally Administered Enoxaparin Ameliorates Acute Colitis by Reducing Macrophage-Associated Inflammatory Responses
Source: PLoS One. 2015 Jul 28;10(7):e0134259. doi: 10.1371/journal.pone.0134259 (PMC4517792; doi:10.1371/journal.pone.0134259)

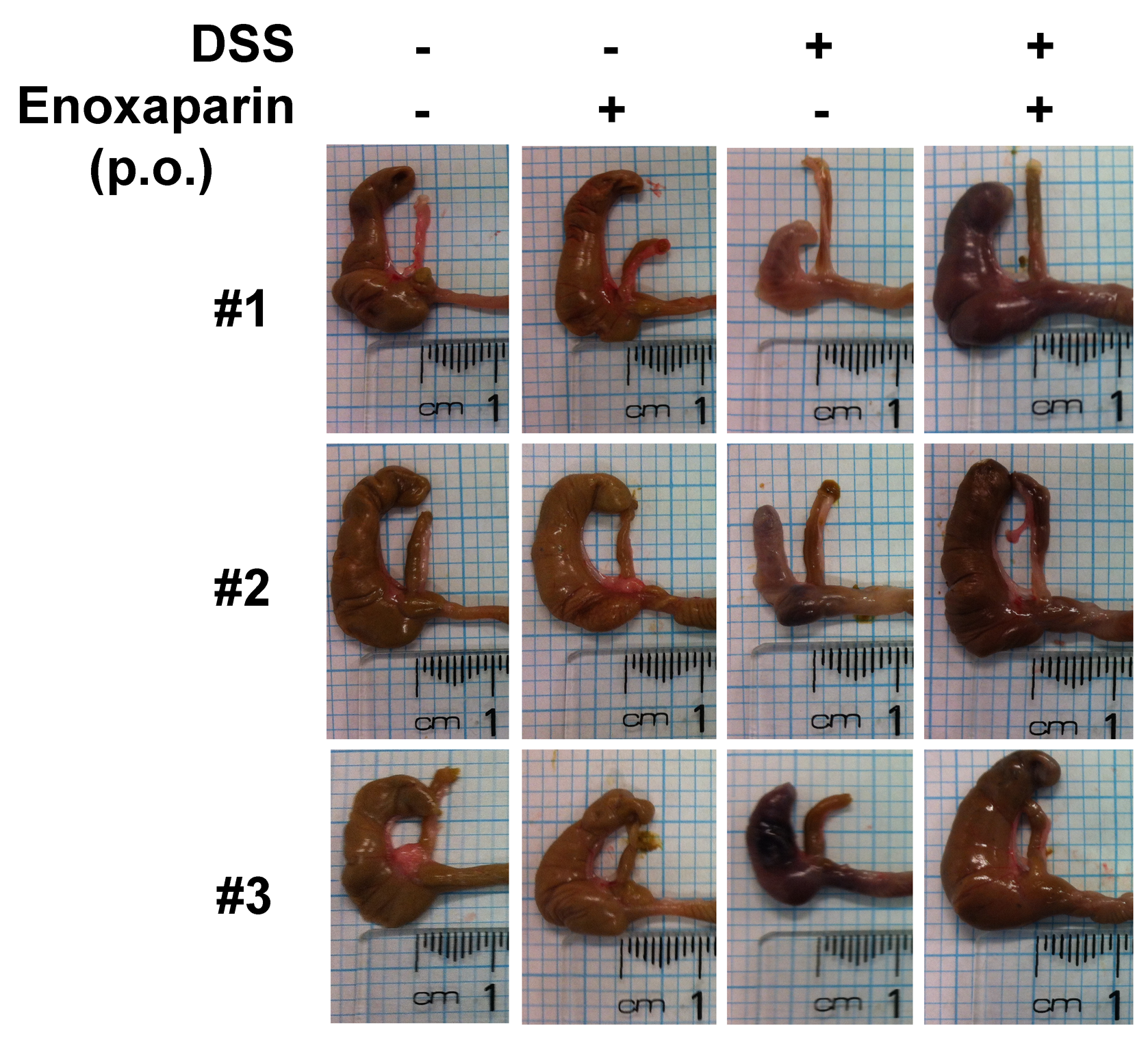

Supplement: S1 Fig — C57BL/6 mice were given 3% w/v DSS in their drinking water from day 1 to day 8. Control mice were given water only. They were treated with or without oral (p.o.) enoxaparin. Cecums were collected on day of termination. Control, C; control with oral enoxaparin, C+OE; untreated colitis, DSS; colitis with oral enoxaparin, DSS+OE. (TIF) [file pone.0134259.s001.tif]

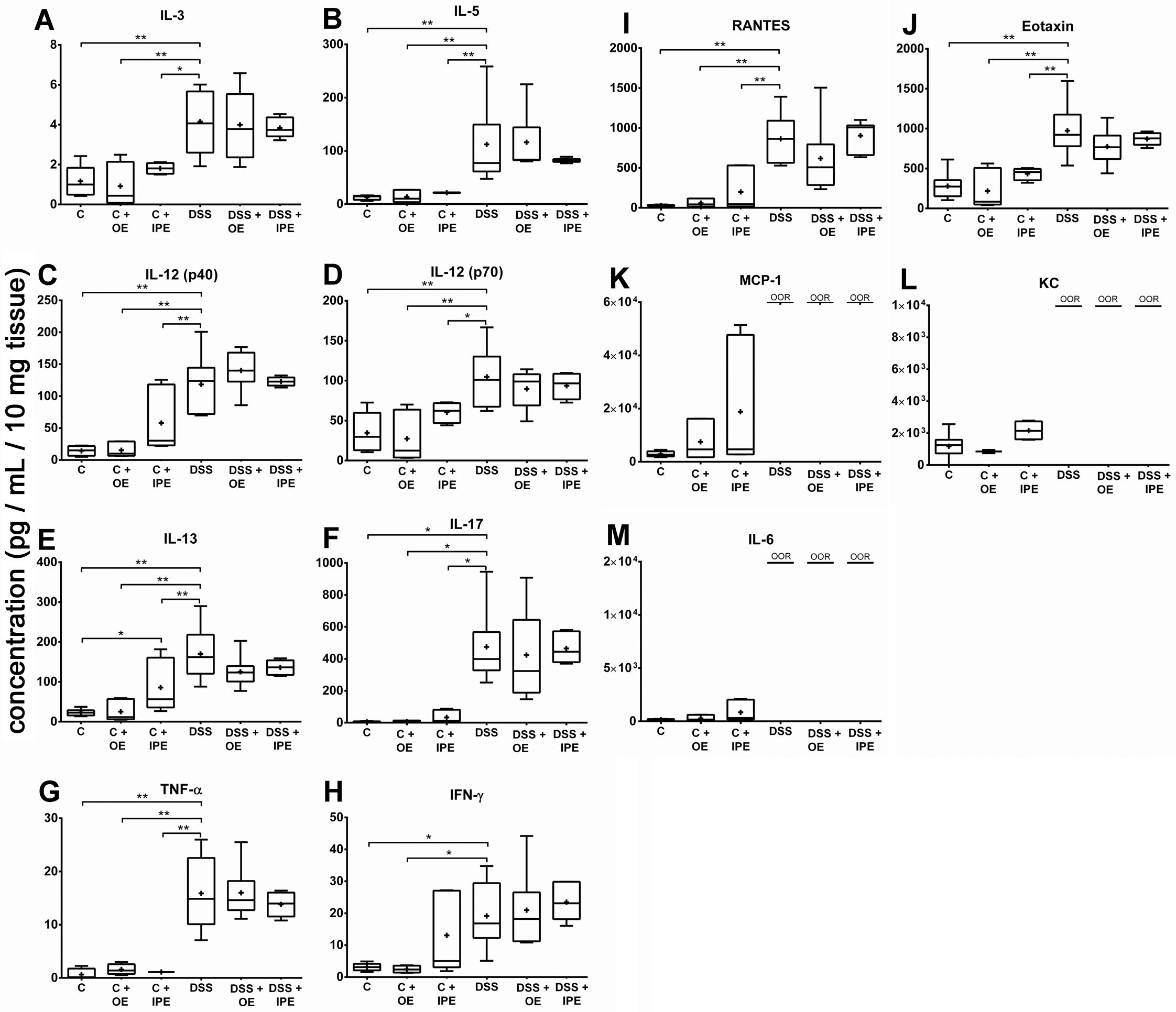

Supplement: S2 Fig — Distal colon tissues of mice were cultured for 24 hours. Supernatants were collected and measured for cytokine levels by using Bio-Plex assay. Cytokine levels in the supernatant were normalized to tissue weight to obtain pg / mL of cytokines/ 10 mg of tissue. Results are expressed as minimum, 25th percentile, median, mean, 75th percentile and maximum of cytokine levels of 3–5 mice. * p < 0.05 and ** p < 0.01. Out of range, OOR; Interleukin, IL; interferon, IFN; keratinocyte-derived chemokine, KC; monocyte chemotactic protein-1, MCP-1; regulated and normal T cells expressed and secreted, RANTES; tumor necrosis factor-α, TNF-α; Control, C; control with oral enoxaparin, C+OE; control with intraperitoneal injection of enoxaparin, C+IPE; untreated colitis, DSS; colitis with oral enoxaparin, DSS+OE; colitis with intraperitoneal injection of enoxaparin, DSS+IPE. (TIF) [file pone.0134259.s002.tif]
